# Freely available genomic datasets for atrial fibrillation research: current resources and analytical pipeline

**DOI:** 10.3389/fgene.2026.1816698

**Published:** 2026-08-24

**Authors:** Meri Gjika, Agnese Sbrollini, Vincenzo Lucio Caputo, Sara Paratico, Emanuela Teresina Locati, Laura Burattini

**Affiliations:** 1 Department of Information Engineering, Università Politecnica delle Marche, Ancona, Italy; 2 Department of Arrhythmology and Electrophysiology, IRCCS Policlinico San Donato, Milan, Italy

**Keywords:** atrial fibrillation, datasets, functional enrichment, gene expression, genomics, Mendeley Data, molecular pathways

## Abstract

Atrial fibrillation (AF) is the most common sustained cardiac arrhythmia, characterized by clinical and genetic heterogeneity. Increasing use of genomics and other omics approaches has driven reliance on publicly available AF datasets to advance biological discovery. Thus, this systematic review aimed to identify freely available genomic AF datasets through Mendeley Data and its interconnected repositories, and to characterize the most common analyses performed on these data. The search was conducted in adherence to the PRISMA 2020 guideline. Nineteen freely available genomic AF datasets were identified: Summary statistics for ‘Biobank-driven genomic discovery yields new insight into atrial fibrillation biology’, hum0014.v8.58qt.v1, AF GWAS in UK Biobank, UK Biobank (Publication 9659), GWAS summary statistics from a 2025 multi-ancestry AF meta-analysis, GSE115574, GSE128188, GSE14975, GSE2240, GSE238242, GSE254133, GSE261170, GSE271748, GSE271839, GSE293813, GSE294456, GSE31821, GSE41177, and GSE79768. The GEO datasets were further examined using differential gene expression, functional enrichment, protein–protein interaction networks, hub gene analysis, microRNA target prediction, and gene clustering, as well as, for the more recently deposited datasets, eQTL colocalization, single-cell/single-nucleus clustering, cell–cell communication analysis, and gene-dosage-dependent transcriptional and electrophysiological profiling. These analyses show some consistency but also considerable heterogeneity in initial conditions, data normalization, and analytical methodological settings. In conclusion, only a limited number of datasets are freely available, so additional, well-characterized and standardized datasets are needed to provide a complete picture of the AF pathology.

## 1 Introduction

Atrial fibrillation (AF) is the most prevalent sustained cardiac arrhythmia (Gutierrez and Chung, 2016; Xu et al., 2016; Antonopoulos et al., 2025), and its global impact is increasing. It has been estimated that by 2060, 17.9 million people in Europe will be affected by AF (Lippi et al., 2020). AF shares a strong epidemiological association with other cardiovascular and systemic diseases, such as heart valve disease, cardioembolic stroke, coronary artery disease, arrhythmogenic and hypertrophic cardiomyopathy, atrial hypertension, diabetes mellitus, and metabolic syndrome. AF is characterized by a low prevalence in individuals under 40 years (<1%), but its occurrence increases with age (Wu et al., 2023; Xin et al., 2023; Lippi et al., 2020; Ruiz-García et al., 2024).

Beyond the growing prevalence and association with comorbidities, AF is also characterized by significant heterogeneity in disease presentation and underlying mechanisms among individuals (Han et al., 2024; Iwamiya et al., 2024; Kalstø et al., 2019; Wijesurendra and Casadei, 2019). AF has both clinical heterogeneities, encompassing differences in patients’ presentation and outcomes, and genetic heterogeneity, reflecting the wide spectrum of inherited and acquired molecular variations that predispose individuals to AF through distinct biological pathways (Kotecha and Piccini, 2015; Christophersen and Ellinor, 2016; Kalstø et al., 2019; Roselli et al., 2020). These diverse origins converge in a shared phenotype, namely the arrhythmia itself, emerging as the final expression of multiple mechanisms (Nattel and Dobrev, 2016; Heijman et al., 2018; Nattel et al., 2020). Thus, the clinical and the genetic heterogeneity of AF reflect a common arrhythmic phenotype arising from multiple converging pathways, including atrial structural abnormalities, electrical remodeling, and autonomic impairment, all of which contribute to the onset and persistence of this arrhythmia (Lubitz et al., 2014; Roden, 2014; Nattel and Dobrev, 2016; Campbell and Wehrens, 2018; Heijman et al., 2018).

In recent years, advancements in genomics technologies have accelerated the understanding of the molecular mechanisms underlying AF (Han et al., 2024; Sweat and Pu, 2024; Zito et al., 2025). Through genome-wide association studies (GWAS), whole-genome sequencing, and transcriptomic analyses, numerous loci and gene variants have been associated with the AF risk (Gutierrez and Chung, 2016; Kornej et al., 2020; Ball et al., 2024; Vad et al., 2024; Yuan et al., 2025). For example, these advancements have associated AF with distinct sets of genes: SCN5A, KCNN3, and KCNQ1, which regulate ion channel activity; PITX2 and ZFHX3, which contribute to atrial structural remodeling, and others in metabolic pathways (Benjamin et al., 2009; Ellinor et al., 2010; Lin et al., 2016; Husser et al., 2017; Roselli et al., 2018). Of note, AF is recognized as a complex disease with a genetic architecture that includes both monogenic and polygenic factors (Gutierrez and Chung, 2016; Andersen et al., 2020; Manoharan et al., 2022; Zhang et al., 2025). Alongside genomic data (i.e., DNA sequences and genetic variants such as SNPs and structural variants (National Institutes of Health, 2024; Offsiteteam, 2024; National Human Genome Research Institute, 2025a), AF research increasingly incorporates multiple omics layers, including transcriptomic data (i.e., RNA expression profiles including mRNA and non-coding RNA (Conesa et al., 2016; National Human Genome Research Institute, 2025b), proteomic data (i.e., protein abundance, modifications, and interactions (Smith and Kelleher, 2013; Aebersold and Mann, 2016), metabolomic data (i.e., small-molecule metabolites reflecting cellular metabolism (Nicholson et al., 1999; Patti et al., 2012), and epigenomic data (i.e., DNA methylation, histone modifications, and chromatin accessibility that regulate gene expression (Jones, 2012; Roadmap Epigenomics Consortium et al., 2015). Each omics type captures different facets of AF pathophysiology, ranging from inherited susceptibility to transcriptional remodeling, protein–protein interactions, metabolic alterations, and regulatory mechanisms (Lam et al., 2006; Stratton and McKinsey, 2016; Christophersen and Ellinor, 2016; Rohun et al., 2024; Roselli et al., 2025).

Recent literature has mainly reported molecular research (Lam et al., 2006; Stratton and McKinsey, 2016; Christophersen and Ellinor, 2016; Rohun et al., 2024; Tang et al., 2024; Choi et al., 2025; Ho et al., 2025; Roselli et al., 2025; Tao et al., 2025), and the increasing use of genomics technologies has led to the appearance of AF-related data resources. These would include not only datasets (i.e., actual raw or processed data files (Merriam-Webster Dictionarya; Oxford English Dictionarya), but also databases (i.e., organized collections of datasets with defined structures, search functions, and curation standards (Oxford English Dictionaryb; Merriam-Webster Dictionaryb), as well as documents that describe and contextualize the data (National Information Standards Organization, 2004; ICPSR, 2012; DDI Alliance and DDI, 2014). These three types of data resources serve different roles: datasets provide analyzable data, databases ensure standardized access and storage, and documents offer interpretative and methodological metadata necessary for reproducibility. Among the available resources for sharing and preserving research datasets, Mendeley Data is an open-access repository that enables researchers to deposit, describe, and disseminate datasets associated with scientific studies. Through its integration with several interconnected repositories and data-sharing platforms, Mendeley Data facilitates the discovery of publicly deposited datasets that can be directly accessed and reused for secondary analyses. In the context of AF genomics, available datasets vary considerably in format (e.g., raw sequencing files, normalized expression matrices, GWAS summary statistics), degree of processing, completeness of metadata, and accessibility (ICPSR, 2012; Wilkinson et al., 2016; GTEx Consortium, 2020; Zhang et al., 2020; Assum et al., 2022; Roselli et al., 2025). These datasets are dispersed across multiple databases and repositories, which differ in their data models, data versioning systems, indexing of metadata, and long-term data preservation policies. Additionally, some datasets are not freely accessible (Chalazan et al., 2023; Christensen et al., 2023; Paludan-Müller et al., 2024; Wijdeveld et al., 2025), significantly limiting the widespread use of AF genomics research. Indeed, this kind of research requires the analysis of genomics datasets, often by advanced statistical and engineering techniques. These include, but are not necessarily limited to, statistical models to assess the significance of individual genetic variants, pathway enrichment analyses to identify biological processes, and machine learning approaches to predict a possible AF risk-based score based on genetic profile (Choi et al., 2019; Kim et al., 2025). Thus, this systematic review aimed to identify AF genomic datasets that were freely available through Mendeley Data and its interconnected repositories, and to identify the most common types of analyses performed on the data.

## 2 Materials and methods

The dataset search was conducted in adherence to the PRISMA 2020 guideline for Systematic Review (Moher et al., 2009; PRISMA, 2020; Higgins et al., 2021; Page et al., 2021). The search was performed by retrieving documents (including abstracts, dataset descriptions, papers, additional and supplementary materials) from open repositories referring to the genomics datasets on AF of interest. Specifically, the datasets search included three phases: (A) document identification, (B) document screening, and (C) document and dataset inclusion. These phases allowed the eligibility assessment in alignment with the PRISMA 2020 framework, which also implies a flow diagram to track the number of documents accepted and excluded at each phase.

### 2.1 Document identification

The search for documents referring to (i.e., describing or utilizing) genomic datasets on AF was conducted through a query on Mendeley Data, a research data repository that contains datasets directly deposited by researchers. Mendeley Data also offers the advantage of making it possible the direct access documents stored on different repositories (like Figshare, Zenodo, Elsevier BV, etc.) with the same query.

The search query contained a combination of two main concepts:

- Eligible documents referring to datasets were related to AF pathology. Identified keywords: “atrial fibrillation” or its abbreviation “AF”.
- Eligible documents referring to datasets were related to genetic data. To get the relevant works, the primarily used keywords were “omics”, “genomics”, and “genetics”. Given the interconnected nature of various omics layers, the following additional related keywords were added: “proteomics”, “metabolomics”, and “multiomics”, even though these additional omics domains did not represent the focus of the analysis.

The keywords were combined using the Boolean operators “AND” if belonging to different concepts, and “OR” if belonging to the same concepts. Thus, the query used was the following:

DATA_TYPE (DATASET) AND (“atrial fibrillation” OR “AF”) AND (“omics” OR “genomics” OR “genetics” OR “proteomics” OR “metabolomics” OR “epigenomics” OR “multiomics”).

A time filter was applied to include only documents published from January 2014 to June 2026, even though in some cases the data could be older. No constraints were applied on the quality or impact of the bibliometric sources; thus, all sources were considered regardless of their citation metrics or journal ranking. Document duplicates, documents not referring to the newest versions of a dataset, and documents not in English were automatically removed by Zotero.

### 2.2 Document screening

The document screening allows acceptance or rejection of the documents in accordance with predefined exclusion criteria in order to include only raw data or processed data. For the purpose of this review and its inclusion criteria, a dataset is considered publicly available if its data files are directly downloadable, and usable without requiring formal application, institutional approval, or restricted access procedures. The screening process was conducted in the following sequential steps:

- Initial screening of documents, title, and metadata.
- Documents retrievability.
- Final screening of the documents, data description or abstract, and full text, if available.

The following exclusion criteria were applied across all screening phases:

- Documents referring to datasets not related to humans.
- Documents referring to datasets concerning pathologies other than AF, unless AF was present as a comorbidity.
- Documents referring to datasets in which the abbreviation “AF” does not refer to atrial fibrillation, but to something else (e.g., allele frequency).
- Documents whose primary focus was on software tools, analytical pipelines, or methodological approaches, rather than on the AF-related omics datasets.
- Documents referring to datasets that include molecular-level analysis but lack genetics content, or in which the focus is not on genetics (e.g., studies focused solely on proteomics, metabolomics, or epigenetics).
- Documents reporting reviews and metadata-only entries without including the primary datasets.
- Documents referring to datasets that required federal or restricted access and were not publicly available.
- Documents referring to datasets that were inaccessible due to a lack of authorization or the unavailability of the full datasets.

### 2.3 Document and dataset inclusion

All the AF genomics datasets referred to in the included documents (i.e., previously identified and screened) were included in the present review. Given the heterogeneous nature of the included datasets, the characterization and critical appraisal were conducted separately for two distinct dataset categories: (1) genetic association studies and GWAS datasets, characterized in terms of data type and public accessibility; and (2) RNA expression and transcriptomic datasets, characterized in terms of microarray or sequencing platform, number of samples, tissue origin, ancestry, surgical conditions, and AF subtype. Specifically, the datasets were used to identify the most common kinds of analysis performed on them.

## 3 Results

**FIGURE 1:**
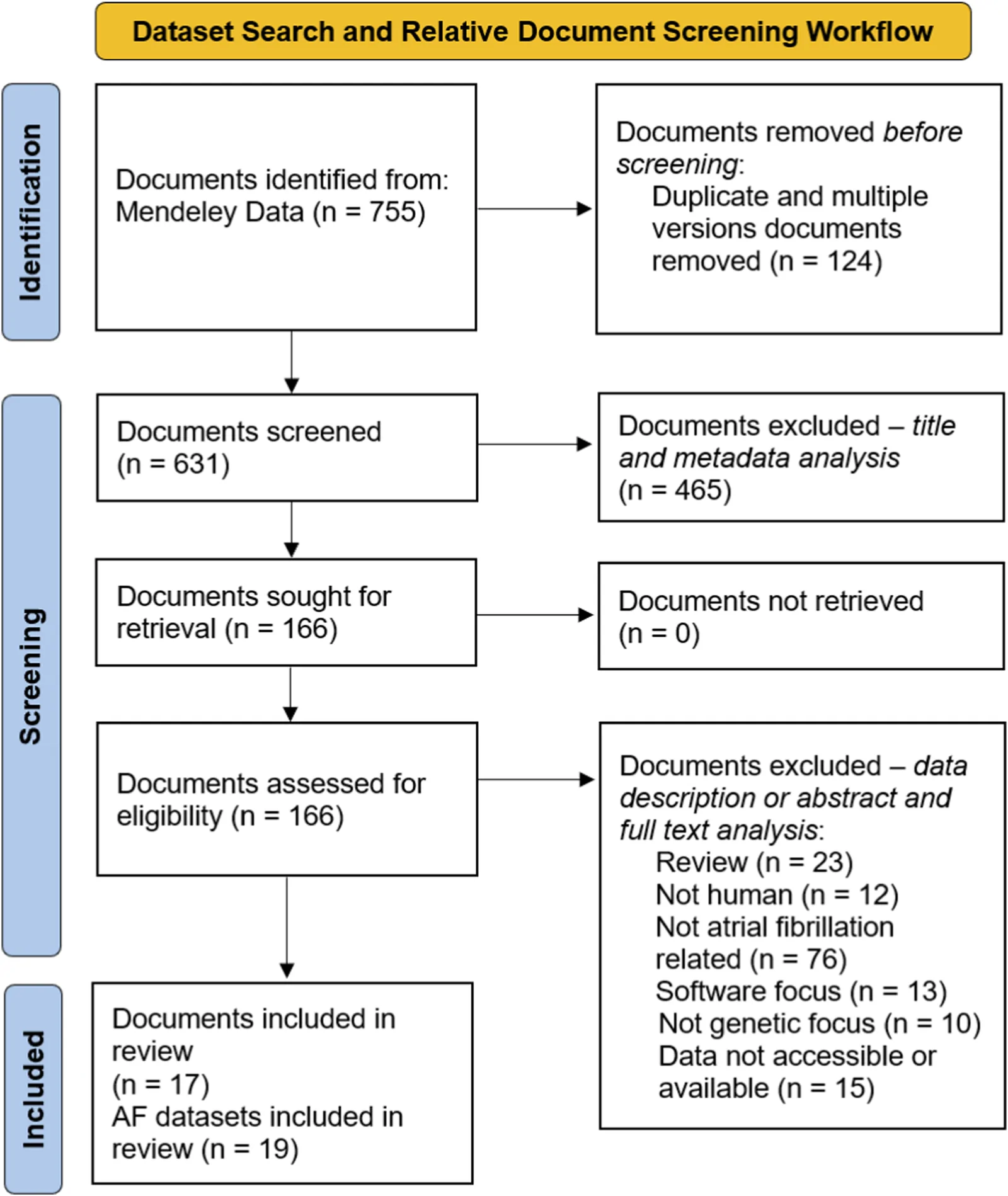
Flowchart of the dataset search strategy (identification, screening, and inclusion phases), as defined by the PRISMA Guidelines.

**FIGURE 2:**
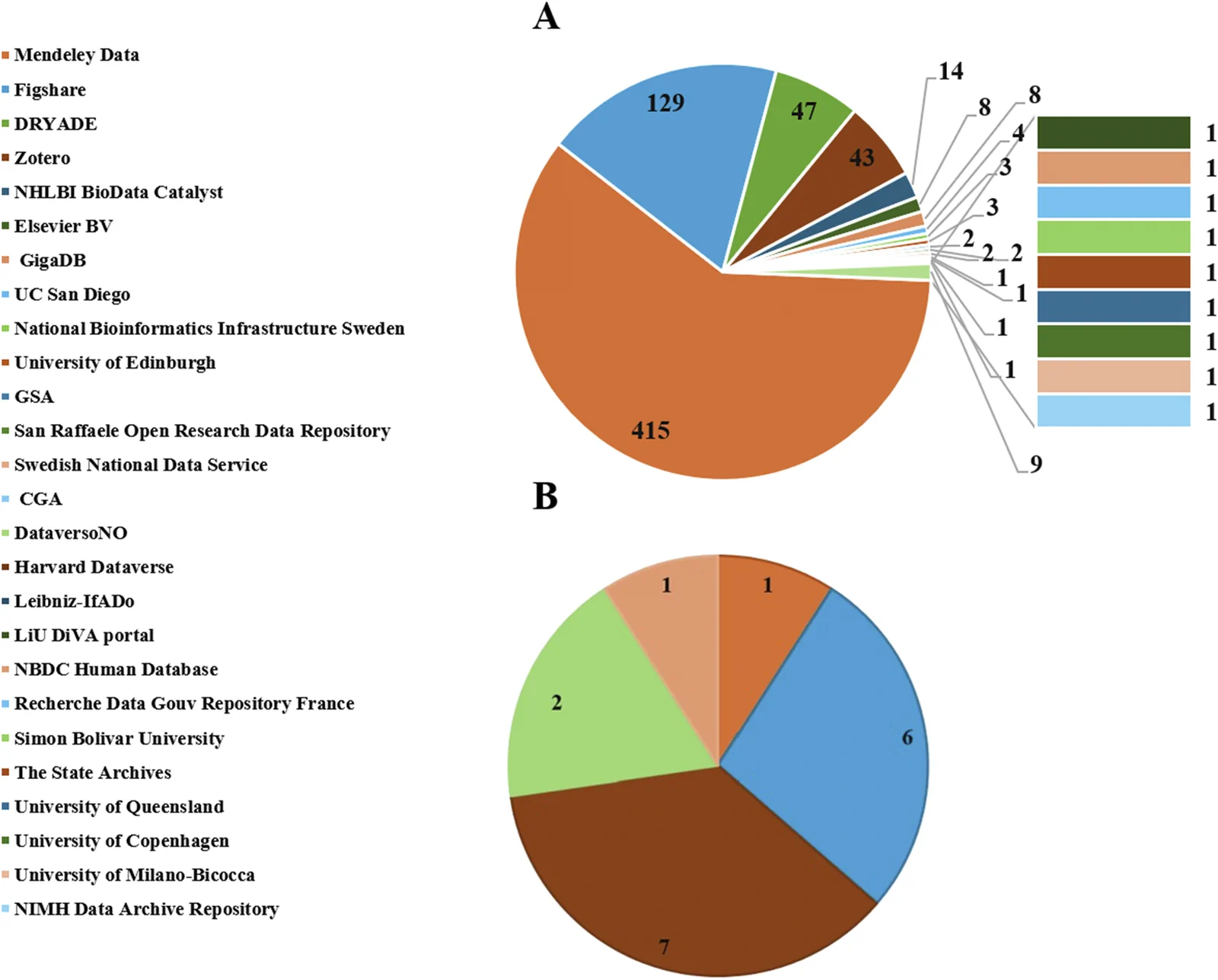
Overview of document distribution across repositories. **(A)** Initial number of documents retrieved per repository. **(B)** Number of documents included after screening.

The results of the application of the datasets search strategy are summarized in the flow diagram depicted in Figure 1, as defined by the PRISMA Guidelines. The search for datasets conducted in Mendeley Data provided 755 initial documents relative to datasets from 27 repositories (Figure 2A): 476 from Mendeley Data, 129 from Figshare, 47 from DRYADE, 47 from Zenodo, 14 from the NHLBI BioData Catalyst, eight from Elsevier BV, eight from GigaDB, four from UC San Diego, three from the National Bioinformatics Infrastructure Sweden, three from University of Edinburgh, two from GSA, two from the San Raffaele Open Research Data Repository, two from the Swedish National Data Service, one from CGA, two from DataversoNO, one from Harvard Dataverse, two from NBDC Human Database, one from Leibniz-IfADo, one from LiU DiVA portal, one from Recherche Data Gouv Repository France, one from Simon Bolivar University, one from The State Archives, one from the University of Queensland, one from the University of Copenhagen, one from University of Milano-Bicocca, and one from NIMH Data Archive Repository. At the end of the procedure, after documents screening, 17 documents from six repositories were included (Figure 2B, Table 1): seven from Zenodo, six from Figshare, one from Mendeley Data, two from DataversoNO, and one from Leibniz-IfADo, all published between 2017 and 2026. The 17 included documents refer to 19 AF datasets (Table 1), namely: Summary statistics for ‘Biobank-driven genomic discovery yields new insight into atrial fibrillation biology’, hum0014.v8.58qt.v1, AF GWAS in UK Biobank, UK Biobank (Publication 9659), GWAS summary statistics from a 2025 multi-ancestry AF meta-analysis (Roselli et al.), GSE115574, GSE128188, GSE14975, GSE2240, GSE238242, GSE254133, GSE261170, GSE271748, GSE271839, GSE293813, GSE294456, GSE31821, GSE41177, and GSE79768.

**TABLE 1:** Summary of selected documents, datasets, and the performed analysis.

Doc. ref.	Publication year	Nation	Repository	AF datasets	Additional datasets (referring to disease)	Performed analysis
Roselli et al. (2025)	2025	Multi-nation	Dataverse NO	GWAS summary statistics from a 2025 multi-ancestry AF meta-analysis	—	—
Nielsen and Willer (2021)	2021	Norway	Dataverse NO	Summary statistics for ‘Biobank-driven genomic discovery yields new insight into atrial fibrillation biology.’	—	—
Chu et al. (2022)	2022	China	figshare	GSE79768	GSE63067(NAFLD)	Identification of DEGs
						Functional Enrichment Analysis of DEGs/Co-DEGs
						Construction of PPI Network of DEGs/Co-DEGs
						Analysis of Hub Genes
						Gene Clustering
						Prediction of microRNA targeting
Kanai et al. (2018)	2018	Japan	Zenodo	hum0014.v8.58qt.v1	—	—
Liu et al. (2021)	2021	China	figshare	GSE2240 GSE14975 GSE41177 GSE79768	—	Identification of DEGs
						Enrichment Analysis, top genes (up/down regulating)
						Gene Clustering
Zheng and He (2021)	2021	France	Leibniz-IfADo	GSE31821	GSE71226(CAD)	Identification of DEGs
						Functional Enrichment Analysis of DEGs/Co-DEGs
						Construction of PPI Network of DEGs/Co-DEGs
						Gene Clustering
van der Harst (2017)	2017	United Kingdom	Mendeley Data	AF GWAS in UK Biobank	CAD GWAS in UK Biobank	—
Assum et al. (2021)	2021	Germany	Zenodo	GSE128188	—	Identification of DEGs
						Functional Enrichment Analysis of DEGs/Co-DEGs
						Gene Clustering
Dai (2021)	2021	China	Zenodo	GSE79768 GSE115574 GSE128188	—	Identification of differentially DEGs
						Functional Enrichment Analysis of DEGs/Co-DEGs
						Enrichment Analysis, top genes (up/down regulating)
						Gene Clustering
Thompson et al. (2022)	2022	United Kingdom	Zenodo	UK Biobank (Publication 9659)	—	—
Zou et al. (2019)	2019	China	figshare	GSE79768	GSE63067(CS)	Identification of DEGs
						Functional Enrichment Analysis of DEGs/Co-DEGs
						Construction of PPI Network of DEGs/Co-DEGs
						Analysis of Hub Genes
						Gene Clustering
						Prediction of microRNA targeting
Leblanc et al. (2024)	2024	Canada	Zenodo	GSE238242 GSE271839	—	Cross-ancestry eQTL fine-mapping
						Single-nucleus multiomic colocalization analysis
Suzuki et al. (2024)	2024	Japan	Zenodo	GSE261170	—	Single-cell/single-nucleus clustering
						Cell–cell communication analysis
van der Maarel et al. (2025)	2025	Netherlands	Zenodo	GSE293813	—	Gene-dosage-dependent transcriptional
						Electrophysiological profiling
Sridhar et al. (2024)	2024	United States	figshare	GSE271748	—	Gene-dosage-dependent transcriptional
						Electrophysiological profiling
Shoda and Emoto (2026)	2026	Japan	figshare	GSE294456	—	—
Zhao (2024)	2024	China	figshare	GSE254133	—	—

AF, Atrial Fibrillation; CAD, Coronary Artery Disease; Co-DEGs, Co-Differentially Expressed Genes; CS, Cardioembolic Stroke; DEGs, Differentially Expressed Genes; Doc. ref., Document of reference; NAFLD, Non-Alcoholic Fatty Liver Disease; PPI, Protein-Protein Interaction.

Five of the included documents refer to datasets containing raw genetic data or GWAS summary statistics: Summary statistics for “Biobank-driven genomic discovery yields new insight into atrial fibrillation biology” (Nielsen and Willer, 2021), hum0014.v8.58qt.v1 (Kanai et al., 2018), AF GWAS in UK Biobank (van der Harst, 2017), UK Biobank (Publication 9659) (Thompson et al., 2022) and GWAS summary statistics from a 2025 multi-ancestry AF meta-analysis (Roselli et al., 2025). These datasets were included in the review as freely downloadable resources but were not subjected to downstream analyses, as summary statistics do not support the types of analyses reviewed here, which require individual-level data files.

**FIGURE 3:**
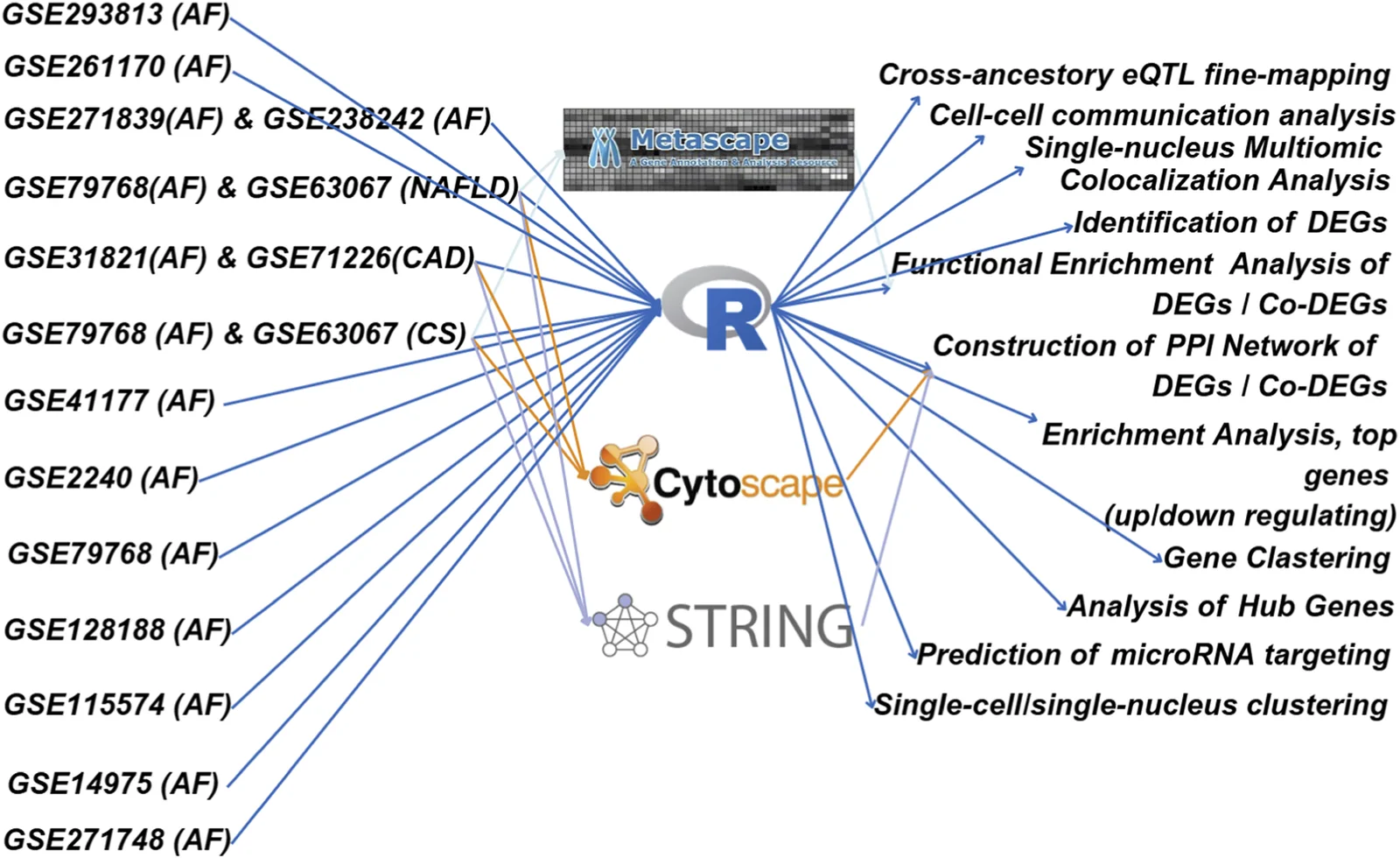
Graphical representation of the analysis performed using selected datasets. The acronym in parentheses after the dataset name indicates the disease the dataset refers to. Arrows indicate the performed analyses, and colours identify the software platform.

Twelve documents correspond to gene expression and genomic datasets, identified by their GEO Series (GSE, i.e., where GEO relates to Gene Expression Omnibus, database repository). Three documents (Dai, 2021; Liu et al., 2021; Leblanc et al., 2024) refer to more than one AF dataset, while two datasets (GSE79768 and GSE128188) are referred to by more than one document (Zou et al., 2019; Assum et al., 2021; Dai, 2021; Liu et al., 2021; Chu et al., 2022). Additionally, four documents refer not only to AF datasets but also to additional datasets relative to other diseases, namely non-alcoholic fatty liver disease (NAFLD (Kotecha and Piccini, 2015), coronary artery disease (CAD) (van der Harst, 2017; Zheng and He, 2021), and cardioembolic stroke (CS) (Zou et al., 2019). Of these twelve documents, six report methodologies relative to some performed data analysis using the pipeline illustrated in Figure 3 (Zou et al., 2019; Assum et al., 2021; Dai, 2021; Liu et al., 2021; Zheng and He, 2021; Chu et al., 2022). Specifically, selected datasets containing pre-processed data were used for downstream analyses, including identification of differentially expressed genes (DEGs), functional enrichment analysis of DEGs and Co-DEGs, construction of protein-protein interaction (PPI) network of DEGs and Co-DEGs, analysis of hub genes, enrichment analysis on top genes (up/down regulating), prediction of microRNA target, and gene clustering. These analyses are illustrated in Figure 3, and for each document, a detailed explanation of the methodology used and of the obtained results for each AF dataset and corresponding comorbidity is reported in Table 2. The remaining six documents (Leblanc et al., 2024; Zhao, 2024; Sridhar et al., 2024; Suzuki et al., 2024; van der Maarel et al., 2025; Shoda and Emoto, 2026) correspond to primary genomic studies, four of which (Leblanc et al., 2024; Sridhar et al., 2024; Suzuki et al., 2024; van der Maarel et al., 2025) applied the following analytic approaches: cross-ancestry eQTL fine-mapping and single-nucleus multiomic colocalization analysis, single-cell/single-nucleus clustering and cell–cell communication analysis, and gene-dosage-dependent transcriptional and electrophysiological profiling, as shown in Figure 3 and Table 2. The remaining two documents (Zhao, 2024; Shoda and Emoto, 2026) lack an associated peer-reviewed publication; consequently, their original analytical methodology could not be verified.

**TABLE 2:** Overview of analyzes, datasets, documents, specific methodologies, and key results obtained for each analysis.

Type of analysis	Doc. ref.	Datasets	Method	Result
*Identification of DEGs*	Chu et al. (2022)	GSE79768 (AF) GSE63067 (NAFLD)	Differential expression analysis was performed in R using the LIMMA package. DEGs were defined as |log_2_FC| > 0.5 and p < 0.05. Genes with log_2_FC > 0 were classified as upregulated, those with log_2_FC < 0 as downregulated, and those with log_2_FC = 0 as unchanged. Co-DEGs were identified across datasets using the Venn Diagram.	523 and 583 DEGs were identified in AF and NAFLD, respectively. Among them, 353 and 488 genes were upregulated, while 215 and 140 were downregulated. 45 genes were co-expressed between the two groups.
	Liu et al. (2021)	GSE2240 (AF) GSE14975 (AF) GSE41177 (AF) GSE79768 (AF)	Differential expression analysis was performed in R using the LIMMA package. DEGs were defined as FDR <0.5 and p < 0.05. Genes with log_2_FC > 0 were classified as upregulated, those with log_2_FC < 0 as downregulated, and those with log_2_FC = 0 as unchanged. Co-DEGs were identified across datasets using the Venn Diagram.	863 DEGs were identified in AF. Among them, 485 were upregulated, while 378 were downregulated.
	Zheng and He (2021)	GSE31821 (AF) GSE71226 (CAD)	Differential expression analysis was performed in R using the GEO2R tool. DEGs were defined as |log_2_FC|>1 and p < 0.05. Genes with log_2_FC > 1 were classified as upregulated, those with log_2_FC < −1 as downregulated, and those with −1≤ log_2_​(FC)≤1 as not significantly changed. Co-DEGs were identified across datasets.	361 and 1932 DEGs were identified in AF and NAFLD, respectively. Among them, 293 and 565 genes were upregulated, while 68 and 1367 were downregulated. 21 genes were co-expressed between the two groups.
	Assum et al. (2021)	GSE128188 (AF)	Differential expression analysis was performed in R using the edgeR package. DEGs were defined as FDR<0.05 and p < 0.05. PCA was used to cluster AF and SR samples across LA and RA.	128 DEGs were identified in LA (52 upregulated, 76 downregulated) and 173 in RA (49 upregulated, 124 downregulated). Overall, 157 genes were downregulated and 90 upregulated in AF, with 43 genes commonly downregulated and 11 commonly upregulated across both atria.
	Dai (2021)	GSE79768 (AF) GSE115574 (AF) GSE128188 (AF)	Differential expression analysis was performed in R using the LIMMA package. DEGs were defined as FDR<0.05 and p < 0.05.	833 DEGs were identified in AF.
	Zou et al. (2019)	GSE79768 (AF) GSE58294 (CS)	Differential expression analysis was performed in R using the LIMMA, affy, and affyPLM packages. DEGs were defined as |log_2_FC|>1, p < 0.05 for AF, and log_2_FC|>1.5, p < 0.05 for CS. Genes with log_2_FC > 0 were classified as upregulated, those with log_2_FC < 0 as downregulated, and those with log_2_FC = 0 as unchanged. Co-DEGs were identified across datasets using the Venn Diagram.	489, 265, 518, and 592 DEGs were identified in AF, CS after 3 h, CS after 5 h, and CS after 24 h, respectively. Among them, 61 were upregulated, while 428 were downregulated for AF only. Four genes were co-expressed between the two groups.
*Functional Enrichment Analysis of DEGs/Co-DEGs*	Chu et al. (2022)	GSE79768 (AF) GSE63067 (NAFLD)	GO and KEGG pathway analyses were performed in R (adjusted p < 0.05). GO terms were classified into BP, CC, and MF categories. Metascape was used for annotation and visualization of enriched terms.	GO and KEGG analyses (clusterProfiler) of co-DEGs showed enrichment in BP terms related to neutrophil activation and immune response; CC terms involving vesicle and granule lumen; and MF terms for carbohydrate binding and immune receptor activity. KEGG pathways were enriched in cytokine–cytokine receptor interaction, IL-17, and chemokine signaling. Metascape confirmed enrichment in immune system processes and locomotion.
	Zheng and He (2021)	GSE31821 (AF) GSE71226 (CAD)	GO and KEGG pathway analyses were performed in R (adjusted p < 0.05). GO terms were classified into BP, CC, and MF categories. DAVID software was used to map the KEGG pathways.	GO and KEGG analyses of DEGs from CAD and AF datasets showed enrichment in BP terms related to transcription regulation (CAD) and extracellular matrix (AF); CC terms involving nucleoplasm (CAD) and exosomes (AF); and MF terms for DNA binding (CAD) and cadherin binding (AF). The 21 common DEGs were enriched in BP terms for blood vessel development, CC terms for cytoplasm, and MF terms for protein binding. KEGG pathways were enriched in mRNA surveillance and ribosome biogenesis (CAD), focal adhesion, MAPK, Wnt, and ECM-receptor interaction pathways (AF), with hematopoietic cell lineage marginally enriched among common DEGs, suggesting a potential link between CAD and AF involving MME (Neprilysin) and TfR1.
	Assum et al. (2021)	GSE128188 (AF)	GO, KEGG pathways, Reactome, and MGI analyses were performed (adjusted p < 0.10).	LA-specific DEGs were enriched in circadian entrainment, nicotine addiction, Wnt signaling, and receptor binding (TGF-β, BMP) pathways. RA-specific DEGs were enriched in extracellular matrix organization, collagen formation/degradation, and clathrin-coated vesicle membrane terms. KEGG results showed distinct enrichment patterns between left and right atrial appendages.
	Dai (2021)	GSE79768 (AF) GSE115574 (AF) GSE128188 (AF)	GSEA v4.0.3 was performed using MSigDB gene sets (c5.bp/cc/mf.v7.0 and c2.cp.kegg.v7.0). Pathways with FDR<0.25 were considered significantly enriched.	GO and KEGG analyses of DEGs showed enrichment in BP terms related to cardiac muscle contraction and ion transport; CC terms involving sarcoplasmic reticulum, myofibril, and plasma membrane; and MF terms for calcium and ATP binding. KEGG pathways were enriched in adrenergic signaling in cardiomyocytes, calcium signaling, and hypertrophic cardiomyopathy.
	Zou et al. (2019)	GSE79768 (AF) GSE58294 (CS)	GO (AmiGO database was used), and KEGG pathway analyses were performed in R (adjusted p < 0.05). GO terms were classified into BP, CC, and MF categories. Metascape was used for annotation and visualization of enriched terms.	GO and KEGG analyses of co-DEGs (ZNF566, PDZK1IP1, ZFHX3, PITX2) showed enrichment in BP terms related to transcription regulation and developmental processes; CC terms involving the nucleus and chromatin; and MF terms for DNA binding and transcription factor activity.
*Construction of PPI Network of DEGs/Co-DEGs*	Chu et al. (2022)	GSE79768 (AF) GSE63067 (NAFLD)	The STRING database was used to construct the PPI network, using a confidence score>0.4 as a cut-off value. Networks were visualized and analyzed with Cytoscape to identify hub genes.	PPI analysis of co-DEGs using STRING identified 26 nodes and 54 edges. Ten hub genes with the highest connectivity scores were CCR2, CCL20, PTPRC, CXCR2, CXCL1, MNDA, S100A9, NCF2, S100A12, and S100A8.
	Zheng and He (2021)	GSE31821 (AF) GSE71226 (CAD)	DEGs were analyzed using STRING (confidence score >0.4) to construct protein–protein interaction networks. Networks were visualized and analyzed with Cytoscape to identify key nodes, functional modules, and hub proteins potentially regulating cellular processes or disease mechanisms.	In CAD, two functional modules were identified with key nodes including U2SURP, LUC7L3, PNN, GYPC, EBP41, and ALAS2. In AF, two modules were found with critical nodes SCD, FABP4, GPAM, TF, CYR61, and VCAN. From the 21 co-DEGs were highlighted MME, TfR1, and LAMP1 were highlighted as interacting proteins potentially linking CAD and AF.
	Zou et al. (2019)	GSE79768 (AF) GSE58294 (CS)	The STRING database was used to construct the PPI network, using a confidence score>0.4 as a cut-off value. Networks were visualized and analyzed with Cytoscape to identify hub genes.	PPI analysis in AF and CS identified 256 and 43 nodes, respectively. Hub genes for AF included LRRK2, CALM1, CXCR4, TLR4, CTNNB1, and CXCR2, while hub genes for stroke included CD19, FGF9, SOX9, GNGT1, and NOG.
*Analysis of Hub Genes*	Chu et al. (2022)	GSE79768 (AF) GSE63067 (NAFLD)	Heatmaps of hub gene (obtained from PPI analysis) expression were constructed in R for both AF and NAFLD. Logistic regression was also performed to check the correlation. To assess the diagnostic potential of these genes, ROC curves were generated, and the AUC was calculated. CTD analysis was conducted.	Hub genes (CXCR2, PTPRC, CCR2, MNDA, NCF2, S100A9, S100A8, S100A12) in AF and NAFLD datasets showed consistent upregulation in patients compared with controls. Logistic regression confirmed significant correlations with AF, and ROC analysis indicated diagnostic potential (AUC 0.7–1.0). CTD analysis linked these hub genes to liver and cardiovascular diseases.
	Zou et al. (2019)	GSE79768 (AF) GSE58294 (CS)	Heatmaps of hub gene (obtained from PPI analysis) expression were constructed in R for both AF and NAFLD. CTD analysis was conducted.	Their expression patterns suggest potential functional relevance in AF and stroke, with AF hub genes mainly involved in inflammatory and NF-κB–related pathways, and stroke hub genes associated with myoblast differentiation and fibroblast growth factor signaling. CTD analysis linked these hub genes to cardiovascular diseases.
*Enrichment Analysis, top genes (up/down regulating)*	Liu et al. (2021)	GSE2240 (AF) GSE14975 (AF) GSE41177 (AF) GSE79768 (AF)	Heatmaps were constructed in R for the top 5 upregulated and downregulated genes across these datasets.	Expression patterns of the top five upregulated and downregulated genes were visualized as heatmaps, showing clear differences between patient and control samples.
	Dai (2021)	GSE79768 (AF) GSE115574 (AF) GSE128188 (AF)	Heatmaps were constructed in R for the top 10 upregulated and downregulated genes across these datasets.	Expression patterns of the top 10 upregulated and downregulated genes were visualized as heatmaps, showing clear differences between patient and control samples.
*Prediction of microRNA targeting*	Chu et al. (2022)	GSE79768 (AF) GSE63067 (NAFLD)	Predicted microRNA–mRNA interactions were explored in R using the DIANA Tools platform. Candidate microRNAs targeting the identified co-DEGs were predicted, with a score threshold greater than or equal to 3	Five high-confidence microRNAs were obtained
	Zou et al. (2019)	GSE79768 (AF) GSE58294 (CS)	Predicted microRNAs targeting Co-DEGs were identified using mirDIP, miRDB, TargetScan, and DIANA tools, and the top 5 microRNAs for each Co-DEG were selected for further analysis	The top 5 microRNAs for each Co-DEG, particularly miR-27a-3p, miR-27b-3p, and miR-494-3p, may serve as potential biomarkers or therapeutic targets for AF-related stroke
*Gene Clustering*	Chu et al. (2022)	GSE79768 (AF) GSE63067 (NAFLD)	Identified Co-DEGs between AF and NAFLD were clustered using hierarchical clustering in R. Heatmaps and dendrograms visualized co-expression patterns, and clusters were reported	Clustering revealed distinct co-expression patterns among Co-DEGs. Enrichment analysis showed clusters associated with immune and inflammatory pathways
	Liu et al. (2021)	GSE2240 (AF) GSE14975 (AF) GSE41177 (AF) GSE79768 (AF)	Identified top 5 DEGs from the enrichment analysis were clustered using hierarchical clustering in R. The heatmap and dendrograms visualized co-expression patterns, and clusters were reported	Two main clusters, with a heatmap and dendrograms highlighting distinct co-expression patterns among the genes
	Zheng and He (2021)	GSE31821 (AF) GSE71226 (CAD)	Identified Co-DEGs between AF and CAD were clustered using hierarchical clustering in R. Heatmaps and dendrograms visualized co-expression patterns, and clusters were reported	Clustering revealed distinct co-expression patterns among Co-DEGs, revealing clusters of potential relevance to AF and CAD.
	Assum et al. (2021)	GSE128188 (AF)	The 50 most significant DEGs were clustered using hierarchical clustering in R. Heatmap and dendrograms visualized co-expression patterns, and clusters were reported	Three main clusters, with a heatmap and dendrograms highlighting distinct co-expression patterns among the genes
	Thompson et al. (2022)	GSE79768 (AF) GSE115574 (AF) GSE128188 (AF)	The top 10 identified DEGs from enrichment analysis were normalized using log2 transformation and z-score scaling, then clustered using hierarchical clustering and k-means clustering in R. Heatmaps and dendrograms visualized co-expression patterns, highlighting clusters of upregulated and downregulated genes, and significant clusters were used to identify functional modules	Two main clusters, with a heatmap and dendrograms highlighting distinct co-expression patterns among the genes
	Zou et al. (2019)	GSE79768 (AF) GSE58294 (CS)	Identified Co-DEGs between AF and CS were clustered using hierarchical clustering in R. Heatmaps and dendrograms visualized co-expression patterns, and clusters were reported	Clustering revealed distinct co-expression patterns among Co-DEGs, highlighting gene clusters of potential relevance to AF and CS.
*Cross-ancestry eQTL fine-mapping*	Leblanc et al. (2024)	GSE238242 (AF) GSE271839 (AF)	eQTL analysis was performed in two independent cohorts of left atrial appendage tissue (CTSN, European-ancestry; Harbin, East Asian-ancestry). Bayesian fine-mapping and colocalization with AF GWAS summary statistics were performed using the coloc R package. Statistical significance was defined as FDR<0.05 for the eQTL studies and p < 5*10^−8^ for the AF GWAS; colocalization was considered significant at H4 posterior probability >0.4	Nine AF loci, colocalization and fine-mapping analyses implicated 14 genes. Candidate causal variants included rs7612445 at *GNB4* and rs242557 at *MAPT*.
*Single-nucleus multiomic colocalization analysis*	Leblanc et al. (2024)	GSE238242 (AF) GSE271839 (AF)	Paired single-nucleus RNA-seq and ATAC-seq data from left atrial appendage were aligned using Cell Ranger ARC and processed in R using Seurat v4 and Signac. Data underwent SCTransform normalization, Harmony batch integration, and scDblFinder doublet removal, then were co-embedded with a reference heart cell atlas to assign cell types and functionally annotate fine-mapped eQTL/GWAS variants. The top prioritized gene was validated via CRISPR inhibition (CRISPRi) in hESC-derived cardiomyocytes	Single-nucleus data functionally annotated the fine-mapped variants, supporting cell-type-specific regulatory roles. CRISPRi repression of the strongest AF-associated eQTL gene, *LINC01629*, resulted in dysregulation of pathways linked to atrial tissue development and the cardiac conduction system
*Single-cell/single-nucleus clustering*	Suzuki et al. (2024)	GSE261170 (AF)	scRNA-seq (CD45^+^ sorted immune cells, n = 8 AF patients) and snRNA-seq (whole tissue, n = 5 AF patients) were processed using Cell Ranger and clustered in R. Differentially expressed genes were assessed using the non-parametric Wilcoxon rank-sum test. GO enrichment of cluster-defining genes was performed with clusterProfiler. Metabolic pathway activity per cluster was assessed with the sc-Metabolism package. RNA velocity was computed in Python using scvelo. Genetic demultiplexing used Cell-SNP with a Snakemake workflow. Data were compared against a published healthy-donor reference dataset (Human Cell Atlas Data Coordination Platform, accession ERP123138)	Five macrophage clusters, three monocyte clusters, five dendritic cell clusters, neutrophils, and mast cells were identified among myeloid cells. Five fibroblast, five endothelial cell, and three cardiomyocyte sub-clusters were also identified. RNA velocity showed classical monocytes differentiating into IL1B + macrophages
*Cell–cell communication analysis*	Suzuki et al. (2024)	GSE261170 (AF)	Cell–cell interaction analysis was performed using the CellChat R package (v1.6.0) on the clustered scRNA-seq/snRNA-seq data, evaluating signaling pathway networks between myeloid cells, fibroblasts, endothelial cells, and cardiomyocytes	IL1B + macrophages send EGF signals to fibroblasts; PDGFB + dendritic cells and TNF + macrophages send PDGF signals to fibroblasts; LYVE1+ resident macrophages send IGF-1 signals to endothelial cells and to SORBS2+ cardiomyocytes; IL1B + macrophages send VEGF and IL1 signals to endothelial cells. *AREG*, an EGF ligand, was the most upregulated gene in AF and was validated as a serum biomarker (elevated in 35.7% of persistent AF patients vs. 0% of controls)
*Gene-dosage-dependent transcriptional profiling*	van der Maarel et al. (2025)	GSE293813 (AF)	Human iPSC-derived pacemaker cardiomyocytes (hiPSC-PCs) were transduced with AAV6 vectors driving PITX2c or mCherry (control) expression under the TNNT2 promoter. scRNA-seq was performed 7 days post-transduction; clusters were assigned based on TNNT2 expression to identify cardiomyocyte populations, and differential gene expression was assessed between PITX2c and control conditions. Exact clustering/DEG software package not confirmed in the available text	Ectopic PITX2c expression caused PITX2 dosage-dependent transcriptional changes in hiPSC-PCs, with loss of pacemaker cell identity mirroring the mild-to-severe pacemaker cell state disruption observed in the *delB* mouse sinus node
	Sridhar et al. (2024)	GSE271748 (AF)	Unbiased RNA-seq was performed separately on lean vs. obese human atrial tissue, control vs. PA-treated hiPSC-aCMs, and PA- vs. PA-GSK-2795039–treated hiPSC-aCMs. Raw FASTQ files were processed using the BioJupies online platform for pathway and upstream transcription factor analysis; Enrichr was used for upstream regulator analysis (GO, KEGG, REACTOME, TRRUST gene sets). Candidate transcription factors were validated by qPCR.	Common upregulated transcription factors across all three comparisons: PPARA, PITX2, ESRRA, TBX5, GATA4, TCF12. Common downregulated: FOSL1, TCF21, FOXM1, FOXE1, KLF4. PITX2 and TBX5 upregulation was confirmed by qPCR in obese human atrial tissue and PA-treated hiPSC-aCMs
*Electrophysiological profiling*	van der Maarel et al. (2025)	GSE293813 (AF)	Electrophysiological characterization of PITX2c-transduced vs. control hiPSC-PCs was performed following scRNA-seq profiling, evaluating pacemaker cell function in relation to PITX2 dosage. Specific recording technique/software not confirmed in the available text	Ectopic PITX2c expression caused PITX2 dosage-dependent electrophysiological changes in hiPSC-PCs, consistent with impaired pacemaker cell function paralleling the sinus node dysfunction and atrial arrhythmia susceptibility observed in the *delB* mouse model
	Sridhar et al. (2024)	GSE271748 (AF)	Whole-cell patch clamping was performed on PA- and PA-GSK-2795039–treated hiPSC-aCMs to record action potential duration (APD10/50/90), total and slow delayed rectifier potassium current (IK, IKs), sodium current (INa), and L-type calcium current (ICa,L). Optical voltage mapping was performed using the IonOptix MyoPacer system with the FluoVolt Membrane Potential Kit. siRNA knockdown of *PITX2* was used to test its causal role in electrophysiological remodeling	PA treatment shortened APD10/50/90 and increased IKs/total IK; these changes were reversed by NOX2 inhibition (PA-GSK-hiPSC-aCMs) and by *PITX2* siRNA knockdown, which abrogated PA-induced APD shortening and reduced maximum upstroke velocity/amplitude, confirming *PITX2* as a mediator of NOX2-driven electrophysiological remodeling

AAV6, Adeno-Associated Virus serotype 6; AF, atrial fibrillation; APD, action potential duration; ATAC-seq, Assay for Transposase-Accessible Chromatin sequencing; AUC, area under the curve; BP, biological process; CAD, coronary artery disease; CC, cellular components; Co-DEGs, Co-differentially expressed Genes; CRISPRi, CRISPR, interference; CS, cardioembolic stroke; CTD, comparative toxicogenomics database; DEGs, Differentially Expressed Genes; Doc. ref., document of reference; eQTL, expression Quantitative Trait Loci; FC, fold change; FDR, false discovery rate; GO, gene ontology; GWAS, Genome-Wide Association Study; hESC, human Embryonic Stem Cell; hiPSC-aCMs, human induced Pluripotent Stem Cell-derived atrial Cardiomyocytes; hiPSC-PCs, human induced Pluripotent Stem Cell-derived Pacemaker cardiomyocytes; ICa,L, L-type Calcium Current; IK, total potassium current; IKs, Slow delayed rectifier potassium current; INa, Sodium Current; LA, left atrium; KECG, kyoto encyclopedia of genes and genomes; MF, molecular functions; MGI, mouse genome informatics; NAFLD, Non-Alcoholic Fatty Liver Disease; NOX2, NADPH, Oxidase 2; PA, palmitic acid; PPI, Protein-Protein Interaction; qPCR, quantitative Polymerase Chain Reaction; RA, right atrium; ROC, receiver operating characteristic; scRNA-seq, single-cell RNA, sequencing; siRNA, small interfering RNA; snRNA-seq, single-nucleus RNA, sequencing; SR, sinus rhythm.

Additionally, Table 3 summarizes the characteristics of the AF datasets used in the analyses, in terms of microarray or sequencing platform, number of samples, and tissue origin. All datasets were derived from *Homo sapiens* atrial tissue, primarily from atrial tissue; exceptions are GSE254133 (peripheral blood mononuclear cells), and GSE271839 and GSE293813 (*in vitro* human embryonic/induced pluripotent stem cell-derived cardiomyocyte models rather than primary patient tissue). Five datasets were generated using the Affymetrix Human Genome U133 Plus 2.0 platform; one was generated using GPL18573-RNA-Seq (SRA); another using the GPL96/GPL97-Affymetrix Human Genome U133A and U133B platform; six using the Illumina NovaSeq 6000 platform for RNA-seq, single-cell RNA-seq, or single-nucleus multiome sequencing; and one using 10X Genomics Chromium single-cell RNA sequencing. Sample sizes ranged from 2 to 32 per dataset, including both AF and sinus rhythm (SR) specimens where a case-control design was used; four of the newly added datasets (GSE271839, GSE293813, GSE271748, and to a lesser extent GSE294456) were not stratified by AF/SR status, being instead organized by *in vitro* experimental condition, obesity/treatment status, or AF-only sampling, respectively. Three datasets have paired samples, including paired left and right atrial (LA and RA) specimens from atrial or appendage tissues (GSE79768), paired LA and RA appendages (GSE128188), and paired LA and RA tissues obtained specifically from patients with mitral regurgitation (GSE115574). Three datasets represented single-site specimens, consisting of isolated RA appendage samples (GSE2240), LA appendage samples (GSE14975), and atrial appendage tissues (GSE31821), joined by three further single-site datasets among the newly added ones: LA appendage tissue (GSE238242), LA tissue obtained via appendectomy (GSE261170), and LA tissue (GSE294456). Only one dataset (GSE41177) represented a region-specific composite sample, consisting of paired LA-pulmonary vein (LA-PV) junction and LA appendage tissues. Tissue samples were obtained during cardiac surgery with cardiopulmonary bypass where reported, with the exception of GSE115574, which was specifically derived from patients undergoing mitral valve surgery in the context of mitral regurgitation and GSE261170, obtained via LA appendectomy. The type of cardiac surgery and specific surgical conditions were not reported for GSE14975, GSE238242, GSE294456, and GSE31821. GSE271748 combined cardiothoracic surgery-derived human atrial tissue with *in vitro* hiPSC-aCMs (not surgically obtained), GSE254133 involved no surgical procedure as AF onset was pharmacologically (crizotinib) induced, and GSE271839 and GSE293813 used entirely *in vitro* stem cell-derived cardiomyocyte models. Population ancestry in the GEO dataset was explicitly reported in four datasets, GSE79768 and GSE128188 (both European) GSE238242 (mixed European and East Asian), and GSE261170 (East Asian), while ancestry was not reported for the remaining datasets. Regarding AF subtype, patients with permanent AF were included in GSE2240, while GSE79768, GSE128188, and GSE238242 included patients with persistent AF; GSE41177 included patients with persistent or permanent AF, and GSE261170 included patients with early and permanent AF. Two datasets involved AF as part of a distinct or comorbid clinical entity: GSE115574 included patients with AF in the context of mitral regurgitation, representing a distinct clinical entity with potentially different underlying pathophysiology, while GSE254133 represented a single case of paroxysmal AF induced by the chemotherapeutic agent crizotinib. GSE294456 compared paroxysmal and non-paroxysmal AF patients directly. AF subtype was not reported for GSE14975 and GSE31821, and was not applicable for GSE271839 and GSE293813 (*in vitro* models) or for GSE271748 (obesity-stratified rather than AF-subtype-stratified).

**TABLE 3:** Summary of GEO datasets of atrial fibrillation, including platform, sample counts, and tissue origin.

AF datasets	Platform	Number of samples (AF/SR)	Tissue origin of the samples	Ancestry	Surgical condition	AF subtype
GSE79768	GPL570 — Affymetrix Human Genome U133 Plus 2.0	26 (14/12)	Paired LA and RA specimens (atria/appendage)	European	Cardiac surgery (cardiopulmonary bypass)	Persistent AF
GSE2240	GPL96/GPL97 — Affymetrix Human Genome U133A and U133B	30 (10/20)	RA appendage	Not reported	Cardiac surgery (cardiopulmonary bypass)	Permanent AF
GSE14975	GPL570 — Affymetrix Human Genome U133 Plus 2.0	10 (5/5)	LA appendage	Not reported	Not reported	Not reported
GSE41177	GPL570 — Affymetrix Human Genome U133 Plus 2.0	32 (32/0)	paired LA-PV junction and LA appendage	Not reported	Cardiac surgery	Persitent/permanent AF
GSE31821	GPL570 — Affymetrix Human Genome U133 Plus 2.0	6 (4/2)	Atrial appendage	Not reported	Not reported	Not reported
GSE128188	GPL18573 — RNA-Seq (SRA)	20 (10/10)	paired LA and RA appendages	European	Cardiac surgery (cardiopulmonary bypass)	Persistent AF
GSE115574	GPL570 — Affymetrix Human Genome U133 Plus 2.0	30 (15/15)	paired LA and RA tissue from patients with MR	Not reported	Mitral valve surgery	AF with mitral valve regurgitation
GSE271839	GLP20301— Illumina HiSeq 4000	6 (N.A. — hESC-CM, LINC01629-KD vs. NTC control, not AF/SR)	hESC-derived cardiomyocytes (*in vitro*)	Not reported	*In vitro* model, not surgical	Not AF-stratified
GSE238242	GPL24676 — Illumina NovaSeq 6000	14 (7/7)	LA appendage	Mixed- European and East Asian	Not reported	Persistent AF
GSE261170	GPL24676 — Illumina NovaSeq 6000	15 (8/7)	LA tissue appendectomy	East Asian (Japan)	LA appendectomy	Early AF and Permanent AF
GSE293813	GPL34284 — Illumina NovaSeq X Plus	Not reported	hiPSC-derived pacemaker cardiomyocytes (*in vitro*)	Not reported	*In vitro* model, not surgical	Not AF-stratified
GSE271748	GPL24676 — Illumina NovaSeq 6000	15 (N. A. — obesity-stratified, not AF/SR)	hiPSC-derived atrial cardiomyocytes (hiPSC-aCMs) + human atrial tissue (HAT)	Not reported	Cardiothoracic surgery (HAT only; hiPSC-aCMs *in vitro*)	Not AF-stratified (obesity/treatment-stratified)
GSE294456	GPL24676 — Illumina NovaSeq 6000	8 (8/0)	LA (CD45^+^ sorted immune cells + whole tissue)	Not reported	Not reported	Paroxysmal vs. non-paroxysmal AF
GSE254133	GPL24676 — Illumina NovaSeq 6000	2 (1/1)	Peripheral blood mononuclear cells	Not reported	Drug-induced AF, not surgical	Paroxysmal AF, crizotinib-induced (single case)

AF, atrial fibrillation; CTSN, cardiothoracic surgical trials network; hiPSC-aCMs, human induced Pluripotent Stem Cell-derived atrial Cardiomyocytes; hiPSC-PCs, human induced Pluripotent Stem Cell-derived Pacemaker Cardiomyocytes; LA, left atrium; LAA, left atrial appendage; MR, mitral regurgitation; N.A., not applicable; PBMC, peripheral blood mononuclear cells; PV, pulmonary vein; RA, right atrium; SR, sinus rhythm.

## 4 Discussion

This study investigated the availability of genomic datasets on atrial fibrillation (AF) through a systematic search conducted using Mendeley Data and related repository indexing services, and reviewed the most common techniques used to further analyze this data. While the heterogeneity of genomic studies is acknowledged in the literature, the systematic documentation and quantification of this heterogeneity across AF-specific datasets, in terms of initial conditions, normalization strategies, and threshold settings, is an important contribution of this work. The systematic search revealed that the number of datasets on the genetics of AF freely available is quite low (19, namely: Summary statistics for “Biobank-driven genomic discovery yields new insight into atrial fibrillation biology”; hum0014.v8.58qt.v1; AF GWAS in UK Biobank; UK Biobank (Publication 9659); GWAS summary statistics from a 2025 multi-ancestry AF meta-analysis; GSE115574; GSE128188; GSE14975; GSE2240; GSE31821; GSE41177; GSE79768; GSE238242; GSE254133; GSE261170; GSE271748; GSE271839; GSE293813; and GSE294456) and distributed on different repositories (Zenodo, Figshare, Mendeley Data, DataversoNO, and Leibniz-IfADo). Only 12 out of 19 FA datasets were further analyzed (Table 2); the kind of analysis differs significantly among documents in terms of aim, used methodology, and used software platform. Nevertheless, the few used methodological approaches were sufficiently well described to allow independent implementation of the analysis procedures and subsequent replication of the performed analysis on the data, which led to substantially equivalent results (unpublished results).

For concerns dataset analysis (Table 2), the studies performing identification of differentially expressed genes were mainly run on the R environment, employed packages like LIMMA (Smyth, 2004) or edgeR (Robinson et al., 2010), and used visualization tools like Venn Diagrams and Volcano Plots. The studies all share a common methodology pipeline, but it is worth noting that the definition and the numerical setting of thresholds varied substantially. For example, the threshold defined as |log_2_FC|, where FC represents the fold change in gene expression between conditions, directly used to discriminate upregulated from downregulated genes (Mariani et al., 2003; McDermaid et al., 2019), ranged from 0.5 to 1.5 among studies; the p-value, used to identify statistical significance, was 0.05 in some studies, while in others the adjusted p-value was used. Notably, these thresholds correspond to markedly different fold-change magnitudes: a |log_2_FC| of 0.585 corresponds to a 1.5-fold change in expression, while a |log_2_FC| of 1.0 corresponds to a 2-fold change. The choice of threshold therefore directly determines both the number and the biological magnitude of genes classified as differentially expressed, with more lenient thresholds capturing subtler expression changes at the cost of an increased false-positive rate, and more stringent thresholds prioritizing specificity and larger-magnitude changes at the cost of sensitivity. In the absence of a universally accepted standard for AF transcriptomic studies, the chosen threshold should be explicitly justified in the Methods relative to the study’s specific aim, and, where feasible, could be addressed by a sensitivity analysis across a range of thresholds to assess the robustness of the resulting gene lists. Such differences likely reflect the specific study design, the available initial information on the dataset, and the initial conditions (e.g., sample type or tissue of origin, patient characteristics, disease stage or progression, and normalization approach of the gene panel), like in the case of comparison studies between AF and other diseases. This heterogeneity had a direct consequence on the number and nature of the identified genes, and a consequence on their classification as upregulated or downregulated. The studies performing functional enrichment analyses all used the Gene Ontology (GO) and Kyoto Encyclopedia of Genes and Genomes (KEGG) frameworks. GO provides a structured vocabulary to describe gene functions across three domains: biological processes, cellular components, and molecular functions (Ashburner et al., 2000; The Gene Ontology Consortium et al., 2023). Instead, KEGG maps genes to known biological pathways, allowing researchers to understand their roles in complex cellular processes (Kanehisa and Goto, 2000; Kanehisa and Goto, 2017). Part of the analysis was conducted in Metascape for a better visualization of the results (Zhou et al., 2019; Yin et al., 2022). The functional enrichment analysis allows the understanding of the coverage of the cellular components, of the molecular functions, and of the biological processes, providing a broader view on the functional perspective, the enrichment analysis, and the sensitivity of the list of differentially expressed genes (DEGs) obtained from the previous step. All studies on the protein-protein interaction (PPI) networks used the STRING database (Szklarczyk et al., 2021; Szklarczyk et al., 2023) with a confidence score greater than 0.4, and Cytoscape was also used for the visualization of the hub genes, which are key molecular nodes that may drive the AF pathology mechanism. Hub gene analyses were conducted and completed, obtaining the receiver operating characteristic (ROC) curve to test the diagnostic power of these genes, representing an attempt to translate the results into possible clinically relevant biomarkers. However, it should be noted that further validation of these genes is needed, and that the limited size of the available datasets remains a major constraint. For the most statistically significant genes, enrichment analysis and visualization of the top upregulated and downregulated genes were conducted in two studies to further explore the functional significance of the differential expression results. The generation of heatmaps across the AF datasets revealed clear clustering between AF and control samples, highlighting consistent transcriptional alterations despite differences in dataset size and analytical approach. Only two studies explored the prediction of microRNA–mRNA interactions to gain insight into post-transcriptional regulation potentially involved in AF, using tools like DIANA (Karagkouni et al., 2018; Vlachos et al., 2015), to further highlight the post-transcriptomic regulation layers of AF pathophysiology. The predicted interactions highlighted several microRNAs that may influence inflammatory, fibrotic, or electrical remodeling pathways relevant to AF pathogenesis. Gene clustering analyses were performed in several studies to further investigate co-expression relationships among DEGs or Co-DEGs. Using hierarchical and, in some cases, k-means clustering in R, the studies visualized expression patterns through heatmaps and dendrograms, revealing distinct gene clusters. These clusters often corresponded to coherent biological modules, with enrichment analyses indicating predominant associations with immune and inflammatory pathways, as well as metabolic and structural remodeling processes relevant to AF pathophysiology. Similarly, some studies applied clustering analyses to delineate gene modules across AF and SR samples, adding further information to the identified co-expression patterns. As with previous analyses, however, these results depend strongly on normalization strategies, dataset quality, and robustness of the clustering parameters. The five more recently deposited datasets instead applied study-specific primary analytical approaches, including eQTL colocalization (GSE271839, GSE238242), single-cell/single-nucleus clustering and cell–cell communication analysis (GSE261170), and gene-dosage-dependent transcriptional and electrophysiological profiling (GSE293813, GSE271748), reflecting a shift toward single-cell resolution and functional variant characterization in more recent AF genomic research.

As for what concerns the datasets characterization (Table 3), the relatively small sample size may limit the generalization power of the findings and the statistical power. Although all datasets were derived from *H. sapiens* atrial tissue (humans), three of the more recently identified datasets originated from peripheral blood mononuclear cells or *in vitro* stem cell-derived cardiomyocyte models rather than primary atrial tissue. Variations in tissue origin, such as LA versus RA appendage or paired atrial regions, may contribute to biological heterogeneity and influence gene expression profiles. Additionally, since most datasets included both AF and SR, interindividual variability related to disease stage, treatment history, and comorbid conditions may further affect the observed patterns. Globally, this review highlighted the strengths and weaknesses of the current analytical approaches. Use of standardized computational environments and well-established toolboxes provides a degree of methodological consistency. However, the lack of agreement on threshold selection and the limited diversity of available datasets constrain generalizability. Expanding the number and scope of publicly accessible AF datasets, together with adopting minimum reporting guidelines and best-practice checklists stratified by study type and tissue source, analogous to existing community standards like Minimum Information About a Microarray Experiment (MIAME) and Minimum Information about a high-throughput Nucleotide Sequencing Experiment (MINSEQE), would be essential to improve cross-study comparability and reproducibility, without constraining the investigator-specific methodological choices that are inherent to transcriptomic research.

This review was specifically scoped to freely accessible AF genomic datasets. It is worth noting, however, that several large-scale controlled-access repositories also have AF-relevant genomic data, including the database of Genotypes and Phenotypes (dbGaP), the European Genome-phenome Archive (EGA), and the Biologic Specimen and Data Repository Information Coordinating Center (BioLINCC), typically linked to large cardiovascular cohorts and clinical trials. A key criticality of these resources is that they require formal data access applications, institutional review, and data use agreements, which can substantially limit their practical usability, particularly for researchers without institutional infrastructure for controlled-access data management. Consequently, they were excluded from the present review.

Tissue selection is a further important consideration for AF genomic research. Atrial appendage tissue, most commonly the left atrial appendage, represents the predominant source among the datasets, reflecting its direct relevance to the atrial electrical and structural remodeling underlying AF, and its availability as surgical waste during cardiac procedures such as valve surgery, cardiopulmonary bypass, or catheter ablation. Ventricular tissue is not present in the datasets as it is less directly informative for AF-specific mechanisms, since AF primarily originates from atria and remodels them rather than ventricular myocardium, although it may be relevant for studying AF-associated ventricular consequences such as tachycardia-induced cardiomyopathy. Peripheral blood-derived sources, including serum, plasma, and peripheral blood mononuclear cells, offer a minimally invasive alternative (as illustrated in the documents relative to the two datasets GSE254133 and GSE294456), but provide only an indirect readout of atrial-specific molecular processes. The appropriate tissue source therefore depends on the specific research question: atrial tissue is generally more suited to mechanistic studies of AF pathophysiology, whereas blood-derived sources are more readily scalable for biomarker-oriented applications requiring larger or more accessible patient cohorts.

Beyond dataset availability, the utility of AF genomic resources for secondary analyses depends critically on the quality and completeness of the associated metadata. It is evident from the review that variability in the reporting of contextual information is a limitation to the reusability of the datasets identified. Contextual information includes, but is not limited to, tissue origin, patient demographics, disease subtype (e.g. paroxysmal vs. persistent AF, or post-operative AF), sample processing conditions, and normalization parameters. Consequently, it is recommended that future studies depositing AF genomic data ensure the open availability of raw data files alongside comprehensive metadata. The following metadata should be included, at minimum: tissue source and laterality (left vs. right atrium), patient ancestry, sample size and group composition, platform and processing details, and disease subtype. Adherence to these principles would substantially increase the value of deposited datasets, enabling their reuse for purposes beyond those of the original study. These additional uses include the development and validation of machine learning models for AF classification, risk stratification, and subtype identification, as well as facilitating cross-study comparisons.

This work showed that, despite the recent publication of studies on the genetics of atrial fibrillation, the availability of freely accessible datasets remains limited. To our knowledge, this is the first systematic review to provide a structured inventory of freely available AF genomic datasets, along with a characterization of the analytical pipelines applied to them, addressing a gap that limits reproducibility and comparability across studies. The methodological focus of this review was intentional, aiming to complement the existing literature with a technical perspective on the computational methodologies and analytical approaches applied to AF genomic datasets. Additionally, if commonly used analysis tools and pipelines provide some consistency, they also show considerable heterogeneity in initial conditions, data normalization, and analytical thresholds that affect the identification of differentially expressed genes and the downstream functional and network analyses. Thus, larger, well-characterized, and standardized datasets are needed to provide a complete picture of the pathology.

## Data Availability

The Publicly available datasets were analyzed in this study. This data can be obtained either by following the procedure indicated in the corresponding reference paper (linked via DOI) to reach the dataset, or through direct download using the provided DOI/link: 10.1038/s41588-024-02072-3 (DataverseNO; GWAS summary statistics from a 2025 multi-ancestry AF meta-analysis) (Roselli et al., 2025); 10.18710/VC2PSH (DataverseNO; summary statistics from ‘Biobank-driven genomic discovery yields new insight into atrial fibrillation biology’) (Nielsen and Willer, 2021); 10.1186/s12920-022-01300-1 (figshare; GEO accessions https://www.ncbi.nlm.nih.gov/geo/query/acc.cgi?acc=GSE79768 and https://www.ncbi.nlm.nih.gov/geo/query/acc.cgi?acc=GSE63067) (Chu et al., 2022); 10.1038/s41588-018-0047-6 (Zenodo; accession hum0014.v8.58qt.v1) (Kanai et al., 2018); 10.1186/s12872-020-01819-0 (figshare; GEO accessions https://www.ncbi.nlm.nih.gov/geo/query/acc.cgi?acc=GSE2240, https://www.ncbi.nlm.nih.gov/geo/query/acc.cgi?acc=GSE14975, https://www.ncbi.nlm.nih.gov/geo/query/acc.cgi?acc=GSE41177, https://www.ncbi.nlm.nih.gov/geo/query/acc.cgi?acc=GSE79768) (Liu et al., 2021); 10.17179/excli2020-3262 (Leibniz-IfADo; GEO accessions https://www.ncbi.nlm.nih.gov/geo/query/acc.cgi?acc=GSE31821 and https://www.ncbi.nlm.nih.gov/geo/query/acc.cgi?acc=GSE71226) (Zheng and He, 2021); 10.17632/2zdd47c94h.1 (Mendeley Data; AF GWAS and CAD GWAS, UK Biobank) (van der Harst, 2017); 10.5281/zenodo.7335298 (Zenodo; GEO accession https://www.ncbi.nlm.nih.gov/geo/query/acc.cgi?acc=GSE128188) (Assum et al., 2021); 10.5281/zenodo.5508929 (Zenodo; GEO accessions https://www.ncbi.nlm.nih.gov/geo/query/acc.cgi?acc=GSE79768, https://www.ncbi.nlm.nih.gov/geo/query/acc.cgi?acc=GSE115574, https://www.ncbi.nlm.nih.gov/geo/query/acc.cgi?acc=GSE128188) (Dai, 2021); 10.5281/zenodo.7034289 (Zenodo; UK Biobank, Publication 9659) (Thompson et al., 2022); 10.1186/s12967-019-1790-x (figshare; GEO accessions https://www.ncbi.nlm.nih.gov/geo/query/acc.cgi?acc=GSE79768 and https://www.ncbi.nlm.nih.gov/geo/query/acc.cgi?acc=GSE63067) (Zou et al., 2019); 10.1016/j.isci.2024.110660 (Zenodo; GEO accessions https://www.ncbi.nlm.nih.gov/geo/query/acc.cgi?acc=GSE238242 and https://www.ncbi.nlm.nih.gov/geo/query/acc.cgi?acc=GSE271839) (Leblanc et al., 2024); 10.1038/s42003-024-07308-w (Zenodo; GEO accession https://www.ncbi.nlm.nih.gov/geo/query/acc.cgi?acc=GSE261170) (Suzuki et al., 2024); 10.1038/s41467-025-66959-3 (Zenodo; GEO accession https://www.ncbi.nlm.nih.gov/geo/query/acc.cgi?acc=GSE293813) (van der Maarel et al., 2025); 10.1172/JCI175447 (figshare; GEO accession https://www.ncbi.nlm.nih.gov/geo/query/acc.cgi?acc=GSE271748) (Sridhar et al., 2024); https://www.ncbi.nlm.nih.gov/geo/query/acc.cgi?acc=GSE294456 (Shoda and Emoto, 2026); https://www.ncbi.nlm.nih.gov/geo/query/acc.cgi?acc=GSE254133 (Zhao, 2024).

## References

[B1] Aebersold R. Mann M. (2016). Mass spectrometric exploration of proteome structure and function. Nature 537, 347–355. 10.1038/nature19949 27629641

[B2] Andersen J. H. Andreasen L. Olesen M. S. (2020). Atrial fibrillation—a complex polygenetic disease. Eur. J. Hum. Genet. 28, 1371–1381. 10.1038/s41431-020-00784-8 33279945 PMC8298566

[B3] Antonopoulos A. S. Kasiakogias A. Kouroutzoglou A. Touloupaki M. Briasoulis A. Papatheodorou E. (2025). Atrial fibrillation burden and management in cardiomyopathies: current evidence and unmet needs. Trends Cardiovasc. Med. 35 (5), 284–293. 10.1016/j.tcm.2025.01.007 39938579

[B4] Ashburner M. Ball C. A. Blake J. A. Botstein D. Butler H. Cherry J. M. (2000). Gene ontology: tool for the unification of biology. Nat. Genet. 25 (1), 25–29. 10.1038/75556 10802651 PMC3037419

[B5] Assum I. Krause J. Zeller T. Schnabel R. B. Heinig M. (2021). “Extended data: tissue-specific multi-omics analysis of atrial fibrillation,”, V3. Geneva, Switzerland (Zenodo): Zenodo. 10.5281/zenodo.7335298 PMC8782899 35064145

[B6] Assum I. Krause J. Scheinhardt M. O. Müller C. Hammer E. Börschel C. S. (2022). Tissue-specific multi-omics analysis of atrial fibrillation. Nat. Commun. 13, 441. 10.1038/s41467-022-27953-1 35064145 PMC8782899

[B7] Ball J. Mahony E. Nehme E. Voskoboinik A. Hogarty J. Dawson L. P. (2024). The burden of atrial fibrillation on emergency medical services: a population-based cohort study. Int. J. Cardiol. 414, 132397. 10.1016/j.ijcard.2024.132397 39084296

[B8] Benjamin E. J. Rice K. M. Arking D. E. Pfeufer A. van Noord C. Smith A. V. (2009). Variants in ZFHX3 are associated with atrial fibrillation in individuals of European ancestry. Nat. Genet. 41, 879–881. 10.1038/ng.416 19597492 PMC2761746

[B9] Campbell H. M. Wehrens X. H. T. (2018). Genetics of atrial fibrillation: an update. Curr. Opin. Cardiol. 33 (3), 304–310. 10.1097/HCO.0000000000000505 29461262 PMC10108376

[B10] Chalazan B. Freeth E. Mohajeri A. Ramanathan K. Bennett M. Walia J. (2023). Genetic testing in monogenic early-onset atrial fibrillation. Eur. J. Hum. Genet. 31 (7), 769–775. 10.1038/s41431-023-01383-z 37217627 PMC10325969

[B11] Choi S. H. Jurgens S. J. Weng L. C. Pirruccello J. P. Roselli C. Chaffin M. (2019). Monogenic and polygenic contributions to atrial fibrillation risk: results from a national biobank. Circ. Res. 126, 200–209. 10.1161/CIRCRESAHA.119.315686 31691645 PMC7007701

[B12] Choi S. H. Jurgens S. J. Xiao L. Hill M. C. Haggerty C. M. Sveinbjörnsson G. (2025). Sequencing in over 50,000 cases identifies coding and structural variation underlying atrial fibrillation risk. Nat. Genet. 57 (3), 548–562. 10.1038/s41588-025-02074-9 40050430 PMC13450804

[B13] Christensen M. A. Bonde A. Sillesen M. (2023). Genetic risk factors for postoperative atrial fibrillation—a nationwide genome-wide association study (GWAS). Front. Cardiovasc. Med. 10, 1040757. 10.3389/fcvm.2023.1040757 37404734 PMC10315824

[B14] Christophersen I. E. Ellinor P. T. (2016). Genetics of atrial fibrillation: from families to genomes. J. Hum. Genet. 61, 61–70. 10.1038/jhg.2015.44 25994868

[B15] Chu Y. Yu F. Wu Y. Yang J. Shi J. Ye T. (2022). Identification of genes and key pathways underlying the pathophysiological association between nonalcoholic fatty liver disease and atrial fibrillation. BMC Med. Genomics 15, 150. 10.1186/s12920-022-01300-1 35790963 PMC9258143

[B16] Conesa A. Madrigal P. Tarazona S. Gomez-Cabrero D. Cervera A. McPherson A. (2016). A survey of best practices for RNA seq data analysis. Genome Biol. 17 (13), 13. 10.1186/s13059-016-0881-8 26813401 PMC4728800

[B17] Dai M. (2021). “The differential inflammatory infiltration and gene expression in left and right atrium in patients with atrial fibrillation,” V1. Geneva, Switzerland (Zenodo): Zenodo. 10.5281/zenodo.5508929

[B18] DDI Alliance, DDI (2014). Data Documentation Initiative – Technical Specification, Version 2.5. Ann Arbor, Michigan, USA (ICPSR, University of Michigan): DDI Alliance.

[B20] Ellinor P. T. Lunetta K. L. Glazer N. L. Pfeufer A. Alonso A. Chung M. K. (2010). Common variants in KCNN3 are associated with lone atrial fibrillation. Nat. Genet. 42, 240–244. 10.1038/ng.537 20173747 PMC2871387

[B21] GTEx Consortium (2020). The GTEx consortium atlas of genetic regulatory effects across human tissues. Science 369 (6509), 1318–1330. 10.1126/science.aaz1776 32913098 PMC7737656

[B22] Gutierrez A. Chung M. K. (2016). Genomics of atrial fibrillation. Curr. Cardiol. Rep. 18 (6), 55. 10.1007/s11886-016-0735-8 27139902 PMC5386395

[B23] Han P. Zhao X. Li X. Geng J. Ni S. Li Q. (2024). Pathophysiology, molecular mechanisms, and genetics of atrial fibrillation. Hum. Cell 38, 14. 10.1007/s13577-024-01145-z 39505800

[B24] Heijman J. Guichard J. B. Dobrev D. Nattel S. (2018). Translational challenges in atrial fibrillation. Circ. Res. 122 (5), 752–773. 10.1161/CIRCRESAHA.117.311081 29496798

[B25] Higgins J. Thomas J. Chandler J. Cumpston M. Li J. Page M. J. (2021). Cochrane Handbook for Systematic Reviews of Interventions. 2nd ed. Chichester, United Kingdom: Wiley.

[B26] Ho K. S. Keefe J. A. Zhao S. Hulsurkar M. M. Jung S. Y. Samee M. A. H. (2025). Multiomic and electrophysiologic analyses reveal that an inherited MRC2 variant causes fibroblast dysfunction and increased atrial fibrillation susceptibility. Am. J. Physiol. Heart Circ. Physiol. 329 (5), H1055–H1071. 10.1152/ajpheart.00452.2025 40983393 PMC12643113

[B27] Husser D. Büttner P. Ueberham L. Dinov B. Sommer P. Arya A. (2017). Association of atrial fibrillation susceptibility genes, atrial fibrillation phenotypes and response to catheter ablation: a gene based analysis of GWAS data. J. Transl. Med. 15, 71. 10.1186/s12967-017-1170-3 28381281 PMC5381139

[B28] ICPSR (2012). Guide to Social Science Data Preparation and Archiving. 6th ed. Ann Arbor, MI: Inter-university Consortium for Political and Social Research.

[B29] Iwamiya S. Ihara K. Nitta G. Sasano T. (2024). Atrial fibrillation and underlying structural and electrophysiological heterogeneity. Int. J. Mol. Sci. 25 (18), 10193. 10.3390/ijms251810193 39337682 PMC11432636

[B30] Jones P. A. (2012). Functions of DNA methylation: islands, start sites, gene bodies and beyond. Nat. Rev. Genet. 13, 484–492. 10.1038/nrg3230 22641018

[B31] Kalstø S. M. Siland J. E. Rienstra M. Christophersen I. E. (2019). Atrial fibrillation genetics update: toward clinical implementation. Front. Cardiovasc. Med. 6, 127. 10.3389/fcvm.2019.00127 31552271 PMC6743416

[B32] Kanai M. Akiyama M. Takahashi A. Matoba N. Momozawa Y. Ikeda M. (2018). Genetic analysis of quantitative traits in the Japanese population links cell types to complex human diseases. Nat. Genet. 50, 390–400. 10.1038/s41588-018-0047-6 29403010

[B33] Kanehisa M. Goto S. (2000). KEGG: kyoto encyclopedia of genes and genomes. Nucleic Acids Res. 28 (1), 27–30. 10.1093/nar/28.1.27 10592173 PMC102409

[B34] Kanehisa M. Goto S. (2017). KEGG: new perspectives on genomes, pathways, diseases and drugs. Nucleic Acids Res. 45 (D1), D353–D361. 10.1093/nar/gkw1092 27899662 PMC5210567

[B35] Karagkouni D. Paraskevopoulou M. D. Chatzopoulos S. Vlachos I. S. Tastsoglou S. Kanellos I. (2018). DIANA TarBase v8: a decade long collection of experimentally supported miRNA–gene interactions. Nucleic Acids Res. 46 (Database issue), D239–D245. 10.1093/nar/gkx1141 29156006 PMC5753203

[B36] Kim M. S. Khurshid S. Kany S. Weng L. C. Urbut S. Roselli C. (2025). Machine learning-based plasma protein risk score improves atrial fibrillation prediction over clinical and genomic models. Circ. Genom. Precis. Med. 18 (4), e004943. 10.1161/CIRCGEN.124.004943 40525300 PMC12257488

[B37] Kornej J. Börschel C. S. Benjamin E. J. Schnabel R. B. (2020). Epidemiology of atrial fibrillation in the 21st century: novel methods and new insights. Circ. Res. 127, 4–20. 10.1161/CIRCRESAHA.120.316340 32716709 PMC7577553

[B38] Kotecha D. Piccini J. P. (2015). Atrial fibrillation in heart failure: what should we do? Eur. Heart J. 36 (46), 3250–3257. 10.1093/eurheartj/ehv513 26419625 PMC4670966

[B39] Lam L. Lind J. Semsarian C. (2006). Application of proteomics in cardiovascular medicine. Int. J. Cardiol. 108 (1), 12–19. 10.1016/j.ijcard.2006.01.002 16466817

[B40] Leblanc F. J. A. Jin X. Kang K. Lee C. J. M. Xu J. Xuan L. (2024). Atrial fibrillation variant-to-gene prioritization through cross-ancestry eQTL and single-nucleus multiomic analyses. iScience 27 (9), 110660. 10.1016/j.isci.2024.110660 39262787 PMC11388022

[B41] Lin H. Mueller-Nurasyid M. Smith A. V. Arking D. E. Barnard J. Bartz T. M. (2016). Gene-gene interaction analysis for atrial fibrillation. Sci. Rep. 6, 35371. 10.1038/srep35371 27824142 PMC5099695

[B42] Lippi G. Sanchis-Gomar F. Cervellin G. (2020). Global epidemiology of atrial fibrillation: an increasing epidemic and public health challenge. Int. J. Stroke 16, 217–221. 10.1177/1747493019897870 31955707

[B43] Liu L. Y. Bai F. Tang Z. Liu N. Liu Q. (2021). Integrative transcriptomic, proteomic, and machine learning approach to identifying feature genes of atrial fibrillation using atrial samples from patients with valvular heart disease. BMC Cardiovasc. Disord. 21, 52. 10.1186/s12872-020-01819-0 33509101 PMC7842070

[B44] Liu Y. (2023). Functional enrichment analysis of differentially expressed genes in Parkinson’s disease by metascape. Front. Genet. 10.3389/fgene.2023.1147819

[B45] Lubitz S. A. Lunetta K. L. Lin H. Arking D. E. Trompet S. Li G. (2014). Novel genetic markers associate with atrial fibrillation risk in Europeans and Japanese. J. Am. Coll. Cardiol. 63 (12), 1200–1210. 10.1016/j.jacc.2013.12.015 24486271 PMC4009240

[B46] Manoharan A. Sambandam R. Ballambattu V. B. (2022). Genetics of atrial fibrillation—an update of recent findings. Mol. Biol. Rep. 49, 8121–8129. 10.1007/s11033-022-07420-2 35587846

[B47] Mariani T. J. Budhraja V. Mecham B. H. Gu C. C. Watson M. A. Sadovsky Y. (2003). A variable fold change threshold determines significance for expression microarrays. FASEB J. 17 (3), 321–323. 10.1096/fj.02-0351fje 12475896

[B48] McDermaid A. (2019). Interpretation of differential gene expression results of RNA-seq: review and recommendations. Brief. Bioinform. 20 (6), 2044–2057. 10.1093/bib/bby044 30099484 PMC6954399

[B49] Merriam-Webster Dictionary “Dataset,”. Springfield, Massachusetts, USA: Merriam-Webster.com. Available online at: https://www.merriam-webster.com/dictionary/dataset (Accessed September 8, 2025).

[B50] Merriam-Webster Dictionary “Database,”. Springfield, Massachusetts, USA: Merriam-Webster.com. Available online at: https://www.merriam-webster.com/dictionary/database (Accessed September 08, 2025).

[B51] Moher D. Tetzlaff J. Altman D. G. , and PRISMA Group (2009). Preferred reporting items for systematic reviews and meta-analyses: the PRISMA statement. PLoS Med. 6 (7), e1000097. 10.1371/journal.pmed.1000097 19621072 PMC2707599

[B52] National Human Genome Research Institute (2025a). Structural Variation. Bethesda, Maryland, USA (NHGRI/NIH): NHGRI Glossary.

[B53] National Human Genome Research Institute (2025b). Transcriptome. Bethesda, Maryland, USA (NHGRI/NIH): NHGRI Glossary.

[B54] National Information Standards Organization (2004). Understanding Metadata. Bethesda, MD: NISO Press.

[B55] National Institutes of Health (2024). Genomic data sharing policy. NIH Office Sci. Policy. Avalible online at: https://grants.nih.gov/policy-and-compliance/policy-topics/sharing-policies/gds (Accessed December 1, 2025).

[B56] Nattel S. Dobrev D. (2016). Electrophysiological and molecular mechanisms of paroxysmal atrial fibrillation. Nat. Rev. Cardiol. 13, 575–590. 10.1038/nrcardio.2016.118 27489190

[B57] Nattel S. Heijman J. Zhou L. Dobrev D. (2020). Molecular basis of atrial fibrillation pathophysiology and therapy: a translational perspective. Circ. Res. 127, 51–72. 10.1161/circresaha.120.316363 32717172 PMC7398486

[B58] Nicholson J. K. Lindon J. C. Holmes E. (1999). Metabonomics’: understanding the metabolic responses of living systems to pathophysiological stimuli *via* multivariate statistical analysis of biological NMR spectroscopic data. Xenobiotica 29 (11), 1181–1189. 10.1080/004982599238047 10598751

[B59] Nielsen J. B. Willer C. J. (2021). Summary statistics for 'Biobank-driven genomic discovery yields new insight into atrial fibrillation biology. DataversoNO V1. 10.18710/VC2PSH PMC6530775 30061737

[B60] Offsiteteam (2024). Understanding Genetic Variations: SNPs, Indels, Structural Variations, and CNVs. Prague, Czech Republic (Offsiteteam company headquarters): Offsiteteam Bioinformatics Blog.

[B61] Oxford English Dictionary. Data Set. Oxford, United Kingdom: Oxford University Press. Available online at: https://www.oed.com/.

[B62] Oxford English dictionary, “Database,” Oxford, United Kingdom: Oxford University Press. Available online at: https://www.oed.com/.

[B63] Page M. J. McKenzie J. E. Bossuyt P. M. Boutron I. Hoffmann T. C. Mulrow C. D. (2021). The PRISMA 2020 statement: an updated guideline for reporting systematic reviews. BMJ 372, n71. 10.1136/bmj.n71 33782057 PMC8005924

[B64] Paludan-Müller C. Vad O. B. Stampe N. K. Diederichsen S. Z. Andreasen L. Monfort L. M. (2024). Atrial fibrillation: age at diagnosis, incident cardiovascular events, and mortality. Eur. Heart. J. 45 (24), 2119–2129. 10.1093/eurheartj/ehae216 38592444 PMC11212824

[B65] Patti G. J. Yanes O. Siuzdak G. (2012). Metabolomics: the apogee of the omics trilogy. Nat. Rev. Mol. Cell Biol. 13, 263–269. 10.1038/nrm3314 22436749 PMC3682684

[B66] PRISMA (2020). PRISMA 2020: preferred reporting items for systematic reviews and meta-analyses. Available online at: https://www.prisma-statement.org/.

[B67] Roadmap Epigenomics Consortium Kundaje A. Meuleman W. Ernst J. Bilenky M. Yen A. (2015). Integrative analysis of 111 reference human epigenomes. Nature 518, 317–330. 10.1038/nature14248 25693563 PMC4530010

[B68] Robinson M. D. McCarthy D. J. Smyth G. K. (2010). edgeR: a bioconductor package for differential expression analysis of digital gene expression data. Bioinformatics 26 (1), 139–140. 10.1093/bioinformatics/btp616 19910308 PMC2796818

[B69] Roden D. M. (2014). Atrial fibrillation genomics: time to take the next step. J. Am. Coll. Cardiol. 63 (12), 1211–1213. 10.1016/j.jacc.2013.12.016 24486270

[B70] Roselli C. Chaffin M. D. Weng L. C. Aeschbacher S. Ahlberg G. Albert C. M. (2018). Multi-ethnic genome-wide association study for atrial fibrillation. Nat. Genet. 50, 1225–1233. 10.1038/s41588-018-0133-9 29892015 PMC6136836

[B19] Rohun J. Dudzik D. Raczak-Gutknecht J. Wabich E. Młodziński K. Markuszewski M. J. (2024). Metabolomics in atrial fibrillation: unlocking novel biomarkers and pathways for diagnosis, prognosis, and personalized treatment. J. Clin. Med. 14 (1), 34. 10.3390/jcm14010034 39797116 PMC11722095

[B71] Roselli C. Rienstra M. Ellinor P. T. (2020). Genetics of atrial fibrillation in 2020: GWAS, genome sequencing, polygenic risk, and beyond. Circ. Res. 127 (1), 21–33. 10.1161/CIRCRESAHA.120.316575 32716721 PMC7388073

[B72] Roselli C. Surakka I. Olesen M. S. Sveinbjornsson G. Marston N. A. Choi S. H. (2025). Meta-analysis of genome-wide associations and polygenic risk prediction for atrial fibrillation in more than 180,000 cases. Nat. Genet. 57 (3), 539–547. 10.1038/s41588-024-02072-3 40050429 PMC12094172

[B73] Ruiz-García A. Serrano-Cumplido A. Escobar-Cervantes C. Arranz-Martínez E. Pallarés-Carratalá V. (2024). Atrial fibrillation prevalence rates and its association with cardiovascular-kidney-metabolic factors: SIMETAP-AF study. Med. Kaunas. 60 (8), 1309. 10.3390/medicina60081309 39202590 PMC11356659

[B74] Shoda M. Emoto T. (2026). “Single-cell TCR profiling in left Atria identifies complement pathway involvement in atrial fibrillation,” in Gene Expression Omnibus, Accession GSE294456. Bethesda, Maryland, USA (Gene Expression Omnibus, NCBI): Kobe University Graduate School of Medicine. Available online at: https://www.ncbi.nlm.nih.gov/geo/query/acc.cgi?acc=GSE294456 (Accessed July 04, 2026).

[B75] Smith L. Kelleher N. L. (2013). Proteoform: a single term describing protein complexity. Nat. Methods 10, 186–187. 10.1038/nmeth.2369 23443629 PMC4114032

[B76] Smyth G. K. (2004). Linear models and empirical Bayes methods for assessing differential expression in microarray experiments. Stat. Appl. Genet. Mol. Biol. 3 (3), Article3. 10.2202/1544-6115.1027 16646809

[B77] Sridhar A. DeSantiago J. Chen H. Pavel M. A. Ly O. Owais A. (2024). Modulation of NOX2 causes obesity-mediated atrial fibrillation. J. Clin. Invest. 134 (18), e175447. 10.1172/JCI175447 39146015 PMC11405042

[B78] Stratton M. S. McKinsey T. A. (2016). Epigenetic regulation of cardiac fibrosis. J. Mol. Cell. Cardiol. 92, 206–213. 10.1016/j.yjmcc.2016.02.011 26876451 PMC4987078

[B79] Suzuki Y. Emoto T. Sato S. Yoshida T. Shoda M. Endoh H. (2024). Left atrial single-cell transcriptomics reveals amphiregulin as a surrogate marker for atrial fibrillation. Commun. Biol. 7, 1601. 10.1038/s42003-024-07308-w 39622943 PMC11612213

[B80] Sweat M. E. Pu W. T. (2024). Genetic and molecular underpinnings of atrial fibrillation. Cardiovasc. Health. 1, 35. 10.1038/s44325-024-00035-5 39867228 PMC11759492

[B81] Szklarczyk D. Gable A. L. Nastou K. C. Lyon D. Kirsch R. Pyysalo S. (2021). The STRING database in 2021: customizable protein–protein networks, and functional characterization of user-uploaded gene/measurement sets. Nucleic Acids Res. 49 (D1), D605–D612. 10.1093/nar/gkaa1074 33237311 PMC7779004

[B82] Szklarczyk D. Kirsch R. Koutrouli M. Nastou K. Mehryary F. Hachilif R. (2023). The STRING database in 2023: protein–protein association networks and functional enrichment analyzes for any sequenced genome of interest. Nucleic Acids Res. 51 (D1), D638–D646. 10.1093/nar/gkac1000 36370105 PMC9825434

[B83] Tang H. Lu K. Wang Y. Shi Y. Ma W. Chen X. (2024). Analyzes of lncRNA and mRNA profiles in recurrent atrial fibrillation after catheter ablation. Eur. J. Med. Res. 29, 244. 10.1186/s40001-024-01799-3 38643140 PMC11031869

[B84] Tao Y. Liu Q. Wang Y. Jiang R. Zhang K. (2025). Genomic data-driven framework for drug target discovery in atrial fibrillation. J. Transl. Med. 23, 1110. 10.1186/s12967-025-07217-4 41094627 PMC12529848

[B85] The Gene Ontology Consortium Balhoff J. Aleksander S. A. Balhoff J. Carbon S. Cherry J. M. Drabkin H. J. (2023). The gene ontology knowledgebase in 2023. Genetics 224, iyad031. 10.1093/genetics/iyad031 36866529 PMC10158837

[B86] Thompson D. Wells D. Selzam S. Peneva I. Moore R. Sharp K. (2022). “UK Biobank release and systematic evaluation of optimised polygenic risk scores for 53 diseases and quantitative traits,”, V2. Geneva, Switzerland (Zenodo): Zenodo. 10.5281/zenodo.7034289

[B87] Vad O. B. Monfort L. M. Paludan-Müller C. Kahnert K. Diederichsen S. Z. Andreasen L. (2024). Rare and common genetic variation underlying atrial fibrillation risk. JAMA Cardiol. 9, 732. 10.1001/jamacardio.2024.1528 38922602 PMC11209175

[B88] van der Harst P. (2017). CAD GWAS in UK Biobank. Amsterdam, Netherlands (Mendeley Data, Elsevier): Mendeley Data, V1. 10.17632/2zdd47c94h.1

[B89] van der Maarel L. E. Mungroo M. R. Mulleners O. J. Rivaud M. R. Verkerk A. O. van Duijvenboden K. (2025). PITX2 dosage-dependent changes in pacemaker cell state underlie sinus node dysfunction and atrial arrhythmias. Nat. Commun. 16, 11197. 10.1038/s41467-025-66959-3 41350282 PMC12712101

[B106] Vlachos I.S. Zagganas K. Paraskevopoulou M.D. Georgakilas G. Karagkouni D. Vergoulis T. (2015). DIANA-miRPath v3.0: deciphering microRNA function with experimental support. Nucleic Acids Res. 43 (W1), W460. Article W466. 10.1093/nar/gkv403 25977294 PMC4489228

[B91] Wijdeveld L. F. J. M. Ajufo E. Challa S. P. Rämö J. T. Wang X. Kany S. (2025). Cardiomyopathy-associated gene variants in atrial fibrillation. JAMA Cardiol. 10 (6), 564–573. 10.1001/jamacardio.2025.0460 40305039 PMC12044542

[B92] Wijesurendra R. S. Casadei B. (2019). Mechanisms of atrial fibrillation. Heart 105, 1860–1867. 10.1136/heartjnl-2018-314267 31444267

[B93] Wilkinson M. D. Dumontier M. Aalbersberg I. J. J. Appleton G. Axton M. Baak A. (2016). The FAIR guiding principles for scientific data management and stewardship. Sci. Data 3, 160018. 10.1038/sdata.2016.18 26978244 PMC4792175

[B94] Wu G. Wu J. Lu Q. Cheng Y. Mei W. (2023). Association between cardiovascular risk factors and atrial fibrillation. Front. Cardiovasc. Med. 10, 1110424. 10.3389/fcvm.2023.1110424 37753167 PMC10518410

[B95] Xin Q. Zhang S. Wang C. Yao S. Yun C. Sun Y. (2023). Prevalence and clinical characteristics of atrial fibrillation in hospitalized patients with coronary artery disease and hypertension: a cross-sectional study from 2008 to 2018. Chin. Med. J. 136 (5), 588–595. 10.1097/CM9.0000000000002471 36914935 PMC10106139

[B96] Xu J. Luc J. G. Y. Phan K. (2016). Atrial fibrillation: review of current treatment strategies. J. Thorac. Dis. 8 (9), E886–E900. 10.21037/jtd.2016.09.13 27747025 PMC5059277

[B105] Yin X. Wang M. Wang W. Chen T. Song G. Niu Y. (2022). Identification of Potential miRNA-mRNA Regulatory Network Contributing to Parkinson’s Disease. Parkinson’s Dis. 2877728. 10.1155/2022/2877728 36105301 PMC9467752

[B97] Yuan S. Chen J. Ruan X. Li Y. Abramowitz S. A. Wang L. (2025). Cross-population GWAS and proteomics improve risk prediction and reveal mechanisms in atrial fibrillation. Nat. Commun. 16, 6426. 10.1038/s41467-025-61720-2 40645996 PMC12254421

[B98] Zhang J. Huang X. Wang X. Gao Y. Liu L. Li Z. (2020). Identification of potential crucial genes in atrial fibrillation: a bioinformatic analysis. BMC Med. Genomics 13, 104. 10.1186/s12920-020-00754-5 32682418 PMC7368672

[B99] Zhang R. Kim M. S. Yin W. Liao S. Zhu X. Yang X. (2025). Contributions of common, rare, and somatic genetic variants to incidence of atrial fibrillation. JAMA Cardiol. 10, 1240. published online Oct. 8. 10.1001/jamacardio.2025.3664 41060636 PMC12509083

[B100] Zhao J. (2024). Single-cell sequencing of PBMC reveals monocyte-mediated inflammation is enhanced in Crizotinib-Induced paroxysmal atrial fibrillation. Gene Expression Omnibus, accession GSE254133, Bethesda, Maryland, USA (Gene Expression Omnibus, NCBI): Southern University of Science and Technology. Available online at: https://www.ncbi.nlm.nih.gov/geo/query/acc.cgi?acc=GSE254133 (Accessed July 04, 2026).

[B101] Zheng Y. He J. Q. (2021). Common differentially expressed genes and pathways correlating both coronary artery disease and atrial fibrillation. EXCLI J. 20, 126–141. 10.17179/excli2020-3262 33564282 PMC7868642

[B102] Zhou Y. Zhou B. Pache L. Chang M. Khodabakhshi A. H. Tanaseichuk O. (2019). Metascape provides a biologist-oriented resource for the analysis of systems-level datasets. Nat. Commun. 10, 1523. 10.1038/s41467-019-09234-6 30944313 PMC6447622

[B103] Zito E. Bianchini L. Sommariva E. Costa M. Forleo G. B. Tondo C. (2025). The genetic mechanisms and pathology of atrial fibrillation: a narrative review. Biomedicines 13, 654. 10.3390/biomedicines13030654 40149630 PMC11940445

[B104] Zou R. Zhang D. Lv L. Shi W. Song Z. Yi B. (2019). Bioinformatic gene analysis for potential biomarkers and therapeutic targets of atrial fibrillation-related stroke. J. Transl. Med. 17, 45. 10.1186/s12967-019-1790-x 30760287 PMC6375208

